# Coumarin-Based Dual Inhibitors of Human Carbonic Anhydrases and Monoamine Oxidases Featuring Amino Acyl and (*Pseudo*)-Dipeptidyl Appendages: In Vitro and Computational Studies

**DOI:** 10.3390/molecules27227884

**Published:** 2022-11-15

**Authors:** Mariangela Agamennone, Marialuigia Fantacuzzi, Simone Carradori, Anél Petzer, Jacobus P. Petzer, Andrea Angeli, Claudiu T. Supuran, Grazia Luisi

**Affiliations:** 1 Department of Pharmacy, “G. d’Annunzio” University of Chieti-Pescara, 66100 Chieti, Italy; 2 Pharmaceutical Chemistry, School of Pharmacy and Centre of Excellence for Pharmaceutical Sciences, North-West University, Potchefstroom 2520, South Africa; 3 Neurofarba Department, Section of Pharmaceutical and Nutraceutical Sciences, University of Florence, Sesto Fiorentino, 50019 Florence, Italy

**Keywords:** amino acyl coumarins, (*pseudo*)-dipeptidyl coumarins, carbonic anhydrases (CAs), monoamine oxidases (MAOs), molecular docking

## Abstract

The involvement of human carbonic anhydrase (hCA) IX/XII in the pathogenesis and progression of many types of cancer is well acknowledged, and more recently human monoamine oxidases (hMAOs) A and B have been found important contributors to tumor development and aggressiveness. With a view of an enzymatic dual-blockade approach, in this investigation, new coumarin-based amino acyl and (*pseudo*)-dipeptidyl derivatives were synthesized and firstly evaluated in vitro for inhibitory activity and selectivity against membrane-bound and cytosolic hCAs (hCA IX/XII over hCA I/II), as well as the hMAOs, to estimate their potential as anticancer agents. *De novo* design of peptide-coumarin conjugates was subsequently carried out and involved the combination of the widely explored coumarin nucleus with the unique biophysical and structural properties of native or modified peptides. All compounds displayed nanomolar inhibitory activities towards membrane-anchored hCAs, whilst they were unable to block the ubiquitous CA I and II isoforms. Structural features pertinent to potent and selective CA inhibitory activity are discussed, and modeling studies were found to support the biological data. Lower potency inhibition of the hMAOs was observed, with most compounds showing preferential inhibition of hMAO-A. The binding of the most potent ligands (**6** and **16**) to the hydrophobic active site of hMAO-A was investigated in an attempt to explain selectivity on the molecular level. Calculated Ligand Efficiency values indicate that compound **6** has the potential to serve as a lead compound for developing innovative anticancer agents based on the dual inhibition strategy. This information may help design new coumarin-based peptide molecules with diverse bioactivities.

## 1. Introduction

Due to its inherent biological relevance and chemical versatility, the coumarin skeleton has attracted significant interest in medicinal and organic chemistry, from as early as its isolation from tonka beans (*coumarou*) in 1820. The coumarin (benzopyran-2-one) scaffold, consisting of a benzene nucleus fused to the six-membered lactone ring or α-pyrone, is an extended electron-rich π-conjugated system with good charge transfer properties [1]. At the biomolecular level, it has been suggested that the binding strength of this aromatic heterocycle to its putative molecular targets is driven by hydrophobic interactions, particularly π-π interactions, as well as hydrogen bonding and dipole-dipole interactions involving the α-pyrone ring. Interestingly, due to the annular strain, which enhances the carbonyl reactivity, the opening of the lactone ring may be observed as a consequence of reactions with essential receptor/enzyme nucleophiles, or enzymes endowed with esterase activity. Furthermore, additional interactions with the specific targets are finely tuned by the different groups at variable positions of the coumarin skeleton. It should not be surprising then that natural and synthetic coumarins have encountered wide pharmaceutical applications such as antimicrobial, anticoagulant, analgesic, antidepressant, and anticancer agents, among others [2,3,4]. Over the past decades, numerous attempts have been made to modify the coumarin nucleus to develop novel biologically active molecules, and several chemical routes, starting from the simple single- or poly-substitution or the fusion with more complicated polycyclic systems to the hybridization approach with different pharmacophores, have been exploited [5].

Nonetheless, coumarins are not only interesting because of their bioactivity profiles, but also because of their metal chelating and optical properties. Particularly, substitution at the 7-position with electron-donating portions, such as hydroxy- or amino-groups, extends the π-π conjugated system of the nucleus, yielding highly fluorescent molecules, which have extensive applications in the investigation of enzyme activity [6,7].

A well-established method for the determination of protease specificity is based on the use of the 7-amino-4-methyl-coumarin (AMC) and similar fluorogenic moieties to label peptide substrates. It came to our attention that these biochemical probes, which are prototypical examples of peptidyl chains directly bound to the amino-coumarin scaffold through an anilido linkage, have not yet been exploited for a wider range of biological activities, such as inhibitory potential towards medicinally important enzymes that are sensitive to the coumarin class, such as carbonic anhydrases (CAs), monoamine oxidases (MAOs), cholinesterases and aromatase amongst others [6,8]. Considering the multitargeting approach in enzyme inhibition, and in order to explore in more detail this class of conjugates, with particular regard to the role of the peptidyl fragment in tuning drug-like properties such as selectivity and lipo/hydrophilicity balance, a small library of amino acyl and unprecedented (*pseudo*)-dipeptidyl coumarins were synthesized and firstly tested as inhibitors of selected CAs and MAOs.

This ongoing interest focused on the CA group, a large family of pH-regulatory metalloenzymes which catalyze the reversible hydration of carbon dioxide to yield bicarbonate and a proton, is encouraged by the crucial role of two membrane-anchored CAs, namely the IX and XII isoforms, in tumorigenesis, as a result of their ability to create an acidic extracellular *milieu* that promotes tumour survival and progression [9,10]. However, the design of specific CA inhibitors (CAIs) that target the tumor-related isoforms (IX and XII) is a challenging task because of the high structural homology among the 15 zinc-dependent human CAs (hCAs) known, whose three-dimensional structures have disclosed only subtle differences at the rim part of the active site cavity and the center region of the protein [11]. Consequently, the strategy to control the selectivity profile of CAIs towards hCA IX and XII over the widely distributed and physiologically relevant hCA I and hCA II isoforms has capitalized on molecules that have been reported to bind the protein in a position that is not essential for the Zn-chelating activity. In this context, coumarins have emerged as isoform-selective inhibitors for the CAs associated with tumorigenesis, by blocking the entrance of the CA active site in their opened, hydrolyzed form (similar to cinnamic acid derivatives). This is the consequence of specific interactions with regions where amino acids variability among the different classes is higher, and distant from the zinc ion.

Due to their central role in the oxidative deamination of neurotransmitters as well as other arylalkyl monoamines of xenobiotic origin, the MAOs represent attractive targets for the treatment of psychiatric and neurodegenerative disorders in humans. These are flavin adenine dinucleotide (FAD)-dependent enzymes attached to the outer membranes of mitochondria in neuronal, glial, and other cells. Human MAOs (hMAOs) consist of two fully characterized isoforms, hMAO-A and hMAO-B, which share approximately 70% sequence identity while differing in substrate specificity, tissue distribution, and inhibitor sensitivity. Selective hMAO-A inhibitors are in clinical use to treat depression and anxiety disorders. hMAO-B selective inhibitors are well-known coadjuvant agents for the treatment of Parkinson’s disease (PD) and Alzheimer’s disease (AD) and may possess neuroprotective properties by reducing oxidative injury due to the age-related increase of this isoform in the brain. In recent works, an exciting new role in cancer progression and metastasis has been highlighted for both MAOs [12], especially MAO-A [13]. The overexpression of the MAO-A isoform has been reported in several types of cancer, including prostate cancer (PCa), lung cancer, glioma, and Hodgkin lymphoma [14,15]. In particular, MAO-A is considered an important contributor in supporting PCa growth and development through epithelial-to-mesenchymal transition (EMT), elevation of ROS, and hypoxia.

A variety of coumarin-based MAO inhibitors have been reported to date, characterized by reversible and often selective inhibition. The chemical properties of the 2*H*-chromen-2-one nucleus explain the binding of coumarin derivatives to the hMAOs, and stabilization by H-bonds and π-π stacking interactions with the two crucial tyrosine residues (Tyr407 and Tyr444 for hMAO-A, and Tyr435 and Tyr398 for hMAO-B) are important factors. The introduction of substituents differing in size, length, and lipophilic or electronic characteristics at variable positions of the coumarin scaffold has been shown to modulate biological activity and isoform selectivity, enabling discrimination between the bipartite (hMAO-B) and monopartite (hMAO-A) substrate/inhibitor binding cavities of the two isoforms.

Pursuing our interest in bioactive coumarins, we have previously reported a chemical and biological investigation of new conjugates in which amino acids were linked to 6-amino-coumarin or AMC. These conjugates exhibited sub-micromolar inhibitory activity towards the membrane-bound hCA XII, with good selectivity ratios if compared to the CA I and CA II human isoforms [16].

**Figure 1:**
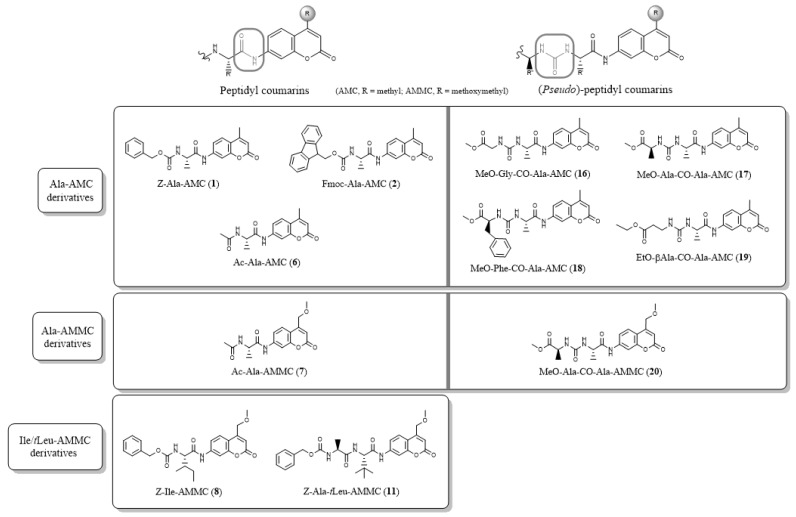
Amino acyl (compounds **1**, **2**, **6**–**8**) and (*pseudo*)-dipeptidyl (**11**, **16**–**20**) derivatives of 4-methyl (AMC)- and 4-methoxymethyl (AMMC)-substituted 7-amino-coumarins.

In the present investigation, the selected coumarin scaffolds, AMC or 7-amino-4-metoxymethyl-coumarin (AMMC), were appropriately functionalized with enantiopure amino acids, to further define the impact of the stereochemistry of the peptide portion on conjugate recognition in the target active sites. The originally designed (*pseudo*)-dipeptidyl coumarin conjugates feature a urea unit placed between the components of the dipeptide portion, i.e., the internal residue, which forms an amide bond with the coumarin 7-amino group, and the terminal amino acid, protected in the ester form. It is worth noting that the central carbonyl of the urea divides the peptide substituent such that the external residue is linked as a retro-isomer, exposing its *C*-terminal functionality (Figure 1). In the field of peptide mimetics, the replacement of regularly arranged peptide bonds with a nonpeptide framework is a well-founded approach to introducing unique chemical and structural properties at the level of the individual backbone fragments [17,18,19,20,21]. This strategy is not only valuable for the rational design of peptide-based substrates and inhibitors for protein targets that will eventually be developed as therapeutics but also provides fundamental insights into the complexity of mutual dynamic interactions involving peptide ligands and their macromolecular acceptors, which leads to pharmacophore optimization and eventually translation of the peptide mimic into a small molecule drug. In this regard, the introduction of a carbonyldiamide (urea) NH-CO-NH linkage such as a peptide bond surrogate is a widely established method that exploits the structural and physico-chemical features of this moiety to improve target affinity and selectivity, as well as stability and bioavailability properties of the resulting *pseudo*-peptide compared to those of the native molecule [22,23,24,25]. Due to the delocalization of the nitrogen lone pair onto the urea carbonyl, which involves both NH functionalities, ureas present with reduced electrophilicity, enhanced chemical stability, and protease resistance relative to amides. The resonance effect gives rise to a more extended planarity of the NH-CO-NH junction and restricts the ω dihedral angle geometry of the two overlapping diamide groups, which results in rigidification of short linear peptide backbones. Furthermore, owing to an augmented H-bond donor capability vs. the amide counterpart, the urea moiety is routinely introduced into peptide backbones to form multiple stable hydrogen bonds with protein/receptor targets and water.

The underlying rationale for designing a peptidyl-extended coumarin scaffold resides in the potential of the chemically heterogeneous (*pseudo*)-peptide portion to create an additional stereo- specific pattern of interactions at the binding site, which is expected to produce improved selectivity between different enzyme isoforms, according to the tail approach [26].

## 2. Chemistry

**Scheme 1:**
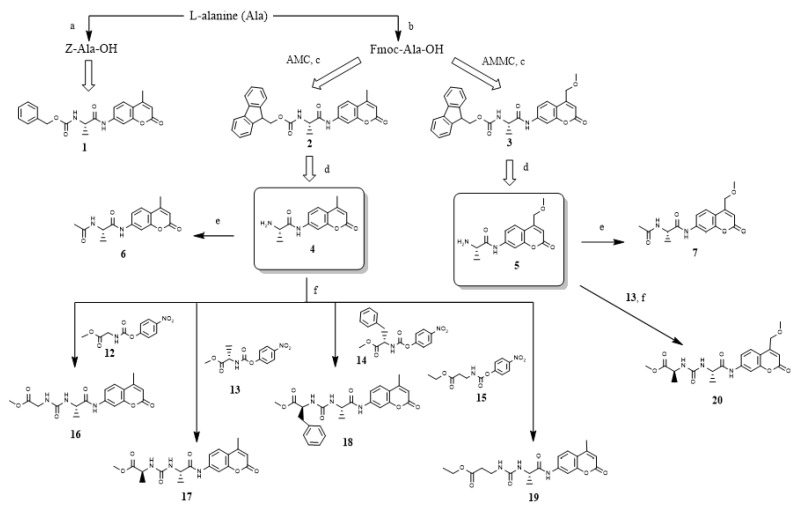
Synthetic route to Ala-AMC/Ala-AMMC derivatives. Reagents and conditions: (**a**) Z-Cl, 1 N NaOH, 0 °C, 2 h, then r.t., 16 h; (**b**) Fmoc-OSu, TEA, H_2_O/THF (2:1), r.t., 4 h; (**c**) IBCF, TEA, dry THF, −10 °C under N_2_, 1 h, then r.t., 24 h; (**d**) DBU, dry DCM, r.t., 15 min; (**e**) Ac_2_O, AcOH, r.t., 21 h; (**f**) DMAP, dry DMF, r.t., 72 h.

**Scheme 2:**
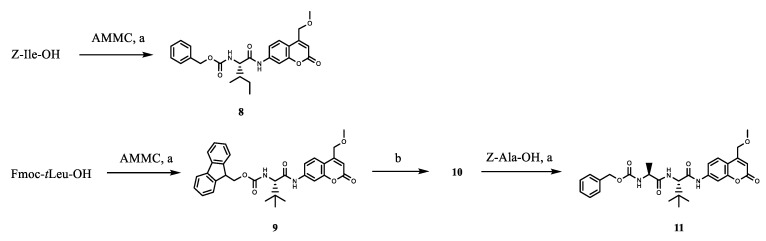
Synthesis of amino acyl and dipeptidyl Ile/tLeu AMMC derivatives **8** and **11**. Reagents and conditions*:* (**a**) IBCF, TEA, dry THF, −10 °C under N_2_, 1 h, then r.t., 20 h; (**b**) DBU, dry DCM, r.t., 15 min.

Amino acyl (compounds **1**, **2**, **6**–**8**) and (*pseudo*)-dipeptidyl coumarins (compounds **11**, **16**–**20**), were synthesized in good overall yields employing solution phase procedures, as outlined in Scheme 1 and Scheme 2. Owing to the poor nucleophilic reactivity of the aromatic amino group, the acylation reactions of coumarins with the conveniently *N*-protected amino acids were preferably conducted by means of the mixed anhydride method. Thus, amino acyl coumarins **8**, **1**, **2**, **3**, and **9** were prepared by reacting the corresponding *N*-capped amino acid with isobutyl cloroformate (IBCF) and triethylamine (TEA) at −10 °C in tetrahydrofuran (THF) under inert atmosphere, followed by the addition of the appropriate coumarin nucleus. After removal of the ice bath, the reaction was completed in 20–24 h and resulted in a crop of the expected conjugate as a brown-red slurry. The work-up of the precipitate and the purification by chromatography and recrystallization gave the expected compounds in satisfactory yields.

Amino acyl coumarins **2** and **3** were the precursors, via 1,8-diazabiciclo[5.4.0]undec-7-ene (DBU)-catalyzed removal of the protecting group under mild conditions [27], of the two key intermediates **4** and **5** required for the assemblage of the most substantial set of coumarin conjugates (amino acyl derivatives **6** and **7**, and (*pseudo*)-peptidyl coumarins **16**–**20**). Acetyl derivatives **6** and **7** were routinely prepared in excellent yields by room temperature reaction of alanyl coumarins **4** and **5**, respectively, with Ac_2_O in acetic acid.

The preparation of dipeptidyl coumarin **11** was accomplished by starting from **9**, which was easily *N*-deprotected by DBU to afford compound **10**. Subsequent acylation was conducted following the mixed anhydride strategy with IBCF-activated Z-Ala-OH, resulting in satisfactory yields of **11**.

The route to the backbone-modified dipeptidyl coumarins **16**–**20**, featuring a urea bridge connecting the two amino acyl moieties, involved the preliminary activations of the external residues as the corresponding *p*-nitro-phenyl carbamates **12**–**15**. They were smoothly obtained in adequate yields by reacting at room temperature for 24 h with the appropriate H-Xaa-OMe/OEt hydrochloride with *p*-nitro-phenyl chloroformate in the presence of pyridine (Py). Subsequent acylation of the suitable alanyl coumarin core (**4**/**5**) with the proper active carbamate (**12**/**13**/**14**/**15**), carried on at room temperature in the presence of catalytic amounts of 4-(dimethylamino)pyridine (DMAP) in *N*,*N*-dimethylformamide (DMF), was completed in 72 h, giving rise to the expected (*pseudo*)-dipeptidyl coumarins **16**–**20** in very good yields.

Final compounds were purified to apparent homogeneity by column chromatography and fully characterized by ^1^H- and ^13^C-NMR spectroscopy. Spectral data are congruent with the expected structures of conjugates. In particular, a diagnostic signal in the range 157.2–158.4 *δ* could be seen in the ^13^C-NMR spectra of the (*pseudo*)-dipeptidyl coumarins (compounds **16**–**20**), consistent with the presence of the urea carbonyl.

## 3. Biochemical Assays and SAR Analysis

### 3.1. CA Inhibition Results

Compounds **1**, **2**, **6**–**8**, **11**, **16**–**20** were initially tested in enzyme inhibition assays against hCA I, II, IX, and XII, and compared with the reference compound acetazolamide (AAZ). The bioactivity results and corresponding selectivity index (SI) between membrane-bound over cytosolic hCAs, as well as of hCA XII compared to hCA IX are reported in Table 1.

Significantly, the whole set of conjugates was found to possess good to excellent inhibitory activity towards the membrane-bound isoforms IX and XII, with *K*_i_ values in the submicromolar to low nanomolar range (*K*_i_ values of 23.4–260.5 nM for hCA IX, and 9.5–336.3 nM for the XII isoform). On the other hand, all the tested compounds were inactive towards the ubiquitous isoenzymes hCA I and II (*K*_i_ ˃ 10.000 nM), thus disclosing very high isoform selectivity with respect to AAZ.

The first series of derivatives (compounds **1**, **2**, **6**–**8**) are the simpler *N*-protected amino acyl-coumarins, with smaller molecular sizes compared to the other tested inhibitors. Apart from **8**, they all possess the alanine residue (compounds **1**, **2**, **6**, and **7**) combined with different *N*-protecting groups (conjugates **1**, **2**, **6**) or variable coumarin scaffolds (AMC for inhibitors **1**, **2**, **6**, and AMMC for **7**). They all exhibit a two-digit nanomolar inhibition against both target isoforms, except inhibitor **1**, which exhibits a *K*_i_ value of 110.0 nM vs. hCA XII. It is worth noting that in the alanyl-AMC/-AMMC main group of derivatives, independently from the coumarin type, the presence of a small acetyl group as *N*-capping substituent gave rise to an efficient and almost comparable inhibition of both hCA IX and hCA XII (as in compounds **6** and **7**, SI = 0.8 and 0.6, respectively). This behavior is not maintained in inhibitors bearing larger and more lipophilic *N*-protecting groups (see compounds **1** and **2**), suggesting that interactions at the protein clefts involving the ligand terminal appendage might be affected by the steric clash and/or electronic issues.

The AMMC-derivative **11** represents the only conjugate with an unmodified dipeptidyl skeleton obtained by incorporating a *t*Leu residue directly linked to the coumarin nucleus. Rather interestingly, **11** displays a significant decrease in inhibitory activity towards both membrane-bound isoforms, particularly towards hCA XII, compared to the above smaller compounds. The activity of **11** may be compared to the shorter, although structurally related, compound **8**, which also possesses a branched, lipophilic side chain. With regard to hCA XII, a large difference in inhibitory activity between compound **8** (still a good inhibitor, with a *K*_i_ value of 41.5 nM), and the approximately nine-fold weaker inhibitor **11** (*K*_i_ = 336.3 nM) was observed. In this case, peptide elongation seems to play a substantial role in reducing binding affinity. In contrast, the difference in the inhibitory constants of **8** and **11** towards hCA IX is smaller (*K*_i_ values of 78.7 nM and 171.5 nM, respectively); it may be suggested that the sterically demanding and highly lipophilic *t*Leu residue, a well-known conformational inducer, perturbs the binding to hCA IX and is here the main contributor to detrimental effects on inhibition.

The most interesting findings were among the (*pseudo*)-dipeptidyl coumarin series (compounds **16**–**20**), with the two low nanomolar hCA XII inhibitors **17** and **19** (*K*_i_ values of 9.6 nM and 9.5 nM, respectively) showing a noticeable gain in selectivity for the hCA XII isoform over the hCA IX (SI hCA XII values of 17.0 for compound **17**; 27.4 for inhibitor **19**). Both compounds possess the Ala-AMC scaffold, linked through the urea bridge to a small, aliphatic residue. It has been argued that the length and/or flexibility of the amino acid backbone, increasing from alanine (see compound **17**) to β-alanine (analogue **19**), may account, albeit partially, for the weaker binding affinity of **19** to hCA IX compared to **17** (*K*_i_ = 260.5 and 163.3 nM, respectively). The relative importance of substitution at the 4-position of the coumarin scaffold (methoxyl vs. the larger methoxymethyl group) for inhibitory activity of the ureic derivatives is exemplified by comparing **17** with the corresponding AMMC counterpart **20**. The latter compound displays a six-fold increase in hCA IX inhibitory activity (*K*_i_ = 27.0 nM against the *K*_i_ value of 163.3 nM for compound **17**). Due to the presence of the conformational constraint imposed by the NH-CO-NH junction, the 4-methoxymethyl group (conjugate **20**) may be forced into a favorable orientation that forms more productive interactions inside the hCA IX active site compared to the 4-methyl homolog, which represents a critical difference in binding to this isoform compared to inhibitor **17**. On the other hand, derivative **17** is an approximate six-fold more efficient inhibitor of hCA XII (*K*_i_ = 9.6 nM) compared to congener **20** (*K*_i_ = 54.7 nM), which demonstrates the opposite effects of the structural and electronic features of the 4-methoxymethyl AMC substituent on hCA XII binding.

It should be noted that this effect is not as pronounced with the smaller inhibitors **6** and **7**, where, conversely, the replacement of AMC for AMMC gives rise to only a very small reduction in both hCA IX and XII inhibitory activities. Here a plausible explanation may be found in the smaller molecular sizes of these ligands (compared to **17** and **20**), which allows the dynamic accommodation of the larger coumarin substituent inside the cleft of both isoforms.

In general, regarding hCA IX inhibitory activity, none of the (*pseudo*)-dipeptidyl derivatives **16**–**20** were found to be superior to the amino acyl coumarins **1**, **2**, **6**–**8**, with compounds **18** and **20** being almost equipotent to **2** and **7**, respectively, and derivatives **16**, **17**, and **19** showing *K*_i_ values that are approximately seven to eleven-fold greater than the value of the best hCA IX inhibitor **6**. In contrast, the hCA XII inhibitory profiles of both series (compounds **1**, **2**, **6**–**8,** and **16**–**20**) partially overlap (with *K*_i_ values ranging from 30.5 to 54.7 nM, except for the three one-digit nanomolar inhibitors **1**, **17** and **19**). It is worth noting that the pairs **2**/**18** and **7**/**20** possess similar inhibitory properties towards either hCA IX or hCA XII, respectively; this may be the result of similarities in structural and physico-chemical properties, such as the presence of the Fmoc/Phe aryl moiety (pair **2**/**18**), or the common AMMC nucleus (pair **7**/**20**).

Overall, the following conclusions may be drawn for this new panel of hCA inhibitors:

- Due to the complete lack of activity towards hCA I and II showing a *K*_i_ ˃ 10,000 nM, the test compounds fulfill the primary goal of selectivity towards membrane-anchored hCAs vs. the cytosolic enzymes;
- All molecules, apart from **1** and **11**, present the structural requisites that are compatible with a potent (one-/two-digit nanomolar *K*_i_ values) hCA XII inhibition;
- hCA IX inhibitory activity is maintained in the low nanomolar range for the smaller amino acyl-coumarin series (compounds **1**, **2**, **6**–**8**), whereas the extension of the (*pseudo*)-peptidyl chain results in up to an eleven-fold reduction of *K*_i_ values when compared to the most effective inhibitor **6** (excluding compounds **18** and **20**, which is similar in potency to **2** and **7**, respectively);
- In the amino acyl coumarin series, hCA inhibitory potency decreases from compounds with small *N*-protecting groups to analogs bearing more extended and lipophilic moieties;
- The general inhibitory profile discloses a significant selectivity towards hCA XII compared to the other isoform that is relevant to cancer; the most remarkable SI values are found in the (*pseudo*)-dipeptidyl coumarin series of derivatives (except **20**), which all share the urea structural motif;
- The replacement of AMC for AMMC in similar structures (pairs **6**/**7** and **17**/**20**) gives rise to unexpected effects, with a slight reduction of activity against both isoforms for the amino acyl-coumarin derivatives, whereas for the (*pseudo*)-dipeptidyl coumarin analogs the same replacement was found to cause a six-fold increase in hCA IX inhibition and a similar reduction in inhibitory activity towards hCA XII;
- The external amino acid also affects activity as observed with the (*pseudo*)-dipeptidyl AMC conjugates. The inhibition potency towards CA IX increases in the order βAla, Gly, Ala, and Phe, suggesting that, in the active site, a larger space is available for additional hydrophobic interactions involving the side chain; Ala and βAla, on the other hand, are the preferred residues for potent hCA XII inhibitory activity, which may be explained by suitable conformational rearrangements into the isoform binding site.

### 3.2. MAO Inhibition Results

Compounds **1**, **2**, **6**–**8**, **11**, **16**–**20** were further evaluated for their ability to inhibit human MAO-A and MAO-B. The inhibition activities are reported in Table 2 (as IC_50_ values) and compared to the reference inhibitors isatin and harmine.

Except for derivative **19**, which was found completely devoid of activity, the tested compounds were able to inhibit either one or both hMAO isoforms. The inhibition potencies were moderate with IC_50_ values in the low micromolar range (IC_50_ = 1.92–79.5 μM). The most potent inhibition was observed for the hMAO-A isoform with inhibitors **6**, **1**, and **16** showing low micromolar IC_50_ values, whilst compounds **7**, **8**, **2**, **11**, **17**, and **18** presented inhibitions in the range 12.0–69.6 μM. The inhibition results suggest that steric size of the coumarin derivatives is an important determinant of MAO-A inhibition. The smallest derivative **6** is thus the most potent MAO-A inhibitor endowed with a 52-fold selectivity for this isoform, while replacement of the 4-methyl with a methoxymethyl (e.g., **7**) and increasing the (*pseudo*)-peptidyl side chain (e.g., **16**) reduce inhibition potency and isoform selectivity. For hMAO-B, only conjugates **7**, **11**, **17**, **18**, and **20** were found to be inhibitors, although with lower potencies compared to hMAO-A (IC_50_ = 45.6–79.5 μM). It is worth noting that compound **7** is the only active compound among amino acyl coumarins **1**, **2**, **6**–**8**, compared to the (*pseudo*)-dipeptidyl series.

### 3.3. Computational Studies

Structure-based computational studies were carried out to obtain more insight into the inhibition activity of studied coumarins toward human CAs and MAOs.

**Scheme 3:**
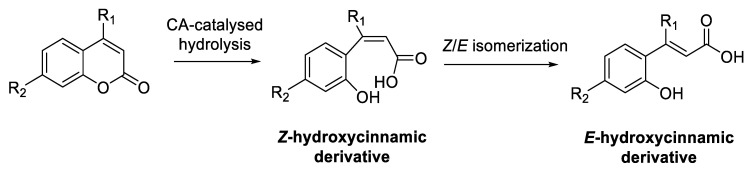
hCA-mediated hydrolysis of coumarin derivatives and successive *Z*/*E* isomerization.

For CAs inhibition, it has been demonstrated that coumarins can be hydrolyzed to *E*/*Z* 2-hydroxycinnamic acid by hCAs because of their esterase activity [28]. The hydrolysis of the coumarin lactone ring directly yields the hydroxycinnamic acid in the *Z* configuration, as observed in the X-ray complex of a naturally occurring coumarin derivative with hCA II (PDB ID: 3F8E) [29]. The isomerization to the *E* form (Scheme 3), which is more stable, has been observed for the simple coumarin in a complex with hCA II (PDB ID: 5BNL) [30]. However, this conversion may not be possible for large ligands in the CA binding site because of their steric hindrance [30]. As we have no experimental evidence on the stabilities (hydrolyzed *E* or *Z*/non-hydrolyzed) of the studied compounds in the binding pocket of hCAs, docking calculations were carried out on the amino-acyl and (*pseudo*)-dipeptidyl coumarins in the non-hydrolyzed and hydrolyzed forms, generating both *E* and *Z* configurations [9]. It may be postulated that, because of their dimensions, the *Z* form could be the most probable active isomer.

In the X-ray complexes of hCA II with coumarin derivatives mentioned above, the hydrolyzed ligands do not coordinate to the zinc ion despite the carboxylate function that could work as the zinc-binding group. Due to the fact that this experimental evidence, we carried out more docking calculations with and without the water molecule as the fourth ligand of the zinc ion. The presence of the water molecule prevents the ligand from binding to the catalytic cation and can serve as an anchoring point for hydrolyzed coumarins. Moreover, to account for the flexibility of the peptide chain and binding to a solvent-exposed region, a previously applied protocol that exploited the SP-peptide docking in Glide [31] was used that allows for expansion of the conformational sampling of the studied ligands [32,33]. The docked poses of ligands into the hCA IX and hCA XII catalytic sites confirm that peptidyl coumarin derivatives in the hydrolyzed form do not bind to the zinc ion, which is also the case when docking was performed without the coordination of the water molecules, as previously demonstrated by De Luca et al. [9]. The ligands occupy the rim portion of the binding site in the region that corresponds to that of hCA II in complex with the hydrolysis products of tetrahydrodehydrogeijerin [6-(1*S*-hydroxy-3-methylbutyl)-7-methoxy-2*H*-chromen-2-one] and the unsubstituted coumarin (PDB IDs: 3F8E and 5BNL, respectively) [29,30], which were selected as reference compounds (Supplementary Materials: Figure S1).

Furthermore, the results show that the docking scores for the hydrolyzed forms are, on average, higher than that obtained for the non-hydrolyzed coumarin derivatives; therefore, we can envision these forms as being responsible for the actual inhibition of hCA IX and hCA XII, particularly when considering their nanomolar potencies [29]. Consequently, the following discussion is mainly centered on ligands in the hydrolyzed form.

**Figure 2:**
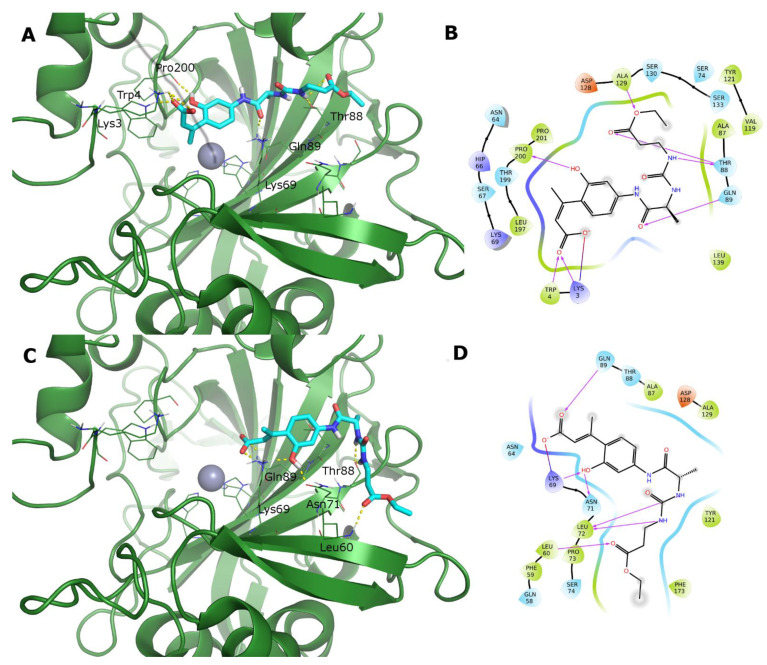
Docked poses of ligand **19** (stick, cyan C atoms) in the *Z* (panel (**A**)) and *E* (panel (**C**)) hydrolyzed forms complexed to hCA XII (dark green cartoon). The most relevant residues are represented as lines. Corresponding 2D ligand interaction diagrams are reported in panels (**B**,**D**), respectively.

Focusing on hCA XII, the binding modes of the *E* and *Z* hydrolyzed forms of compound **19** are reported (Figure 2). A detailed analysis of the ligand docked poses in the hCA XII binding site shows different geometries for the two configurations: the *cis* form has a more extended binding geometry which hampers access to the zinc ion and establishes mainly polar contacts with the Lys3, Trp4, Pro200, Thr88, Gln89, and Ala129 residues. The ligand in the *trans* hydrolyzed form presents a bent geometry interacting with Leu60, Lys69, Asn71, Leu72, and Gln89. The docked geometries and contacts described here are conserved among almost all ligands, as demonstrated by the Interaction diagrams (Figure S2).

**Figure 3:**
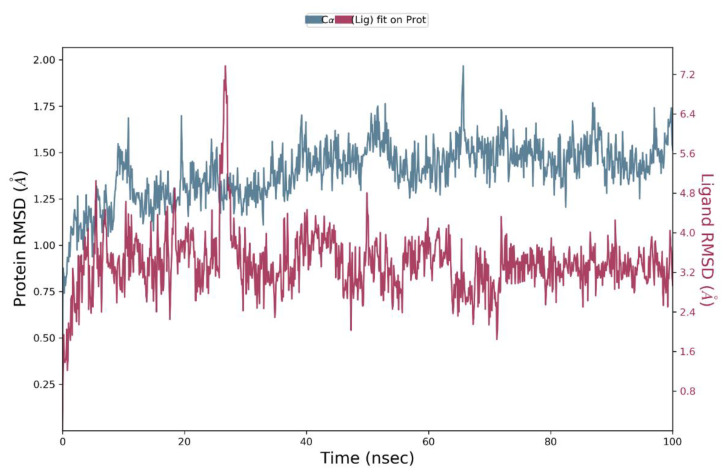
RMSD evolution during the MD simulation of the protein (light blue, calculated on the C-alpha, left *Y*-axis) and the ligand (compound **19**) (purple, calculated on all atoms, right *Y*-axis).

To further validate our prediction, the most conserved docked pose of compound **19**, as the hydrolyzed *Z*-form, in the hCA XII binding site was submitted to MD simulation to verify its stability and reliability. A 100 ns MD simulation was carried out where the protein maintains a stable conformation (maximum RMSD 2.00 Å, Figure 3), while the ligand slightly moves from the above-described docked pose and reaches a stable geometry throughout the simulation as well, apart from a short conformational rearrangement at 27.4 ns where the ligand urea function loses its interaction with Thr88 to form two intramolecular H-bonds with the β-Ala carbonyl group.

**Figure 4:**
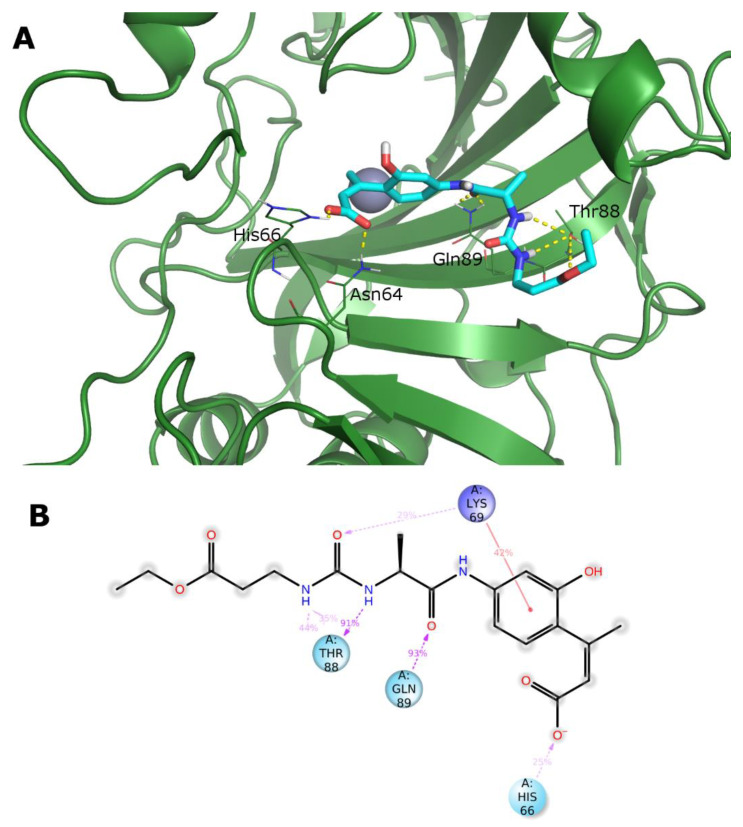
(**A**) Depiction of the binding geometry explored by ligand **19** in a representative frame of the MD simulation. (**B**) 2D representation of most conserved ligand-protein interactions with an indication of the persistence (%) along the simulation.

The alignment of representative geometries obtained from MD frames clustering, reported in Figure S7, further highlights the stable binding of compound **19** in the hCA XII binding region. One of the most representative geometries is depicted in Figure 4A. In the docking pose, the ligand establishes an ionic interaction between the carboxylic acid and Lys3 (Figure 2A). Embedding the complex in the box of explicit water molecules while running the simulation, H-bonding between the two urea NHs and Thr88 is favored.

The carboxylic function can alternatively bind to His66, in a more extended conformation or to Lys69 in a bent geometry. Additionally, the latter residue forms a relatively stable cation-π contact with the aromatic ring of the hydroxycinnamic acid and an H-bond with urea carbonyl oxygen. The high stability of H-bonding contacts to Thr88 and Gln89 makes these latter the anchoring residues with a persistence of 90% during the simulation (Figure 4B). The stability of these H-bonds which involves the urea internal NH and the other polar contacts formed by the distal NH and the carbonyl oxygen, can contribute to explaining the better activity profile shown by (*pseudo*)-dipeptidyl derivatives with respect to amino acyl derivatives toward hCA XII.

The MD simulation confirms that, as already observed for the docked compounds, almost all ligand-protein contacts are polar (Figure S3), and the ligand is solvent-exposed. Assuming that all studied ligands share this behavior, this observation can provide an explanation for the improved activity profile shown by smaller and less hydrophobic ligands, while the presence of large and hydrophobic sidechains or protecting groups are less favored.

**Figure 5:**
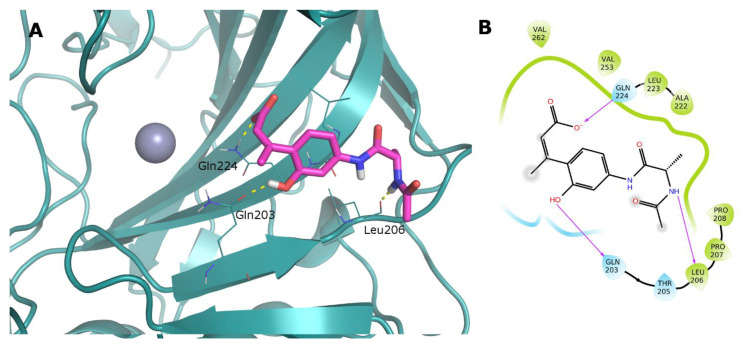
(**A**) Docked pose of ligand **6** (stick, magenta C atoms) in the hydrolyzed *Z* form in the hCA IX binding site (cartoon, dark cyan). (**B**) Corresponding 2D ligand interaction diagram.

The above docking protocol was applied to study the binding of peptidyl coumarins to hCA IX as well. The docked poses of the ligands share some similarities to those retrieved in hCA XII: the hydrolyzed forms of the ligands do not bind the zinc ion and occupy the corresponding region of the binding site. Additionally, in this instance, the binding geometry is well conserved among hCA IX ligands (data not shown). The docked pose of the most active ligand **6** is reported in Figure 5. The terminal amidic NH forms a hydrogen bond with the carbonyl oxygen of Leu206 (corresponding to Thr88 of hCA XII), the carboxylic function binds the terminal NH_2_ of Gln224 (that corresponds to Gln89 of hCA XII), while the phenolic OH is H-bound to the terminal C=O of Gln203 (corresponding to Lys69 of hCA XII).

It is known from the literature that the binding regions reported for coumarins are the most variable among hCAs, which can be exploited to obtain selective inhibitors [34,35,36,37,38]. To further confirm this observation and to obtain a more detailed explanation of the activity profiles of the studied compounds, the sequence similarity of the hCA I, II, IX, and XII isoforms was evaluated. The multiple sequence alignment based on the whole structure superposition, carried out in Maestro, provided an identity/similarity percentage of 40/54% for hCA IX, 36/52% for hCA II, and 37/53% for hCA I using the hCA XII as the reference.

When limiting the comparison to hCA XII residues that are mainly involved in the ligand binding (Lys3, Trp4, Lys57-Phe59, Asn64, His66, Lys69, Asn71, Leu72, Ala87, Thr88, and Gln89 in hCA XII), the identity/similarity further reduces to 33/33% and 31/31% for hCA II and hCA I, respectively, which is noteworthy since this region is formed by most diverse residues for the cytosolic hCAs. Similarity remains the same (38/54%) for hCA IX, which provides a possible explanation for the observed high selectivity (Selectivity Index hCA I or II/hCA XII > 30). Furthermore, we focused on the four most stable interactions retrieved from the MD simulation, which involved His66, Ly69, Thr88, and Gln89 in the hCA XII binding site. It has been verified that His66 and Gln89 are conserved among the studied isoforms, while hCA XII Lys69 is substituted by His67 in hCA I, Asn67 in hCA II and Gln203 in CA IX, and hCA XII Thr88 by Phe91 in hCA I, Ile91 in hCA II, and Leu223 in hCA IX. Therefore, these residues could be responsible for isoform selectivity. Residue variation between hCA XII and hCA IX, moreover, can explain the selectivity profile shown by ligands **16**, **17**, and **19** toward hCA IX; in fact, MD simulation indicates that the promising activity of these compounds as inhibitors of hCA XII is mainly due to the H-bonding between the ligand urea function and protein residue, Thr88. Therefore, the substitution of Thr88 with Leu206 eliminates the productive contact observed in the hCA XII binding site.

With regard to MAOs, molecular docking calculations were performed with human MAO-A and MAO-B in an attempt to explain the binding modes of the most potent and selective compounds toward hMAO-A (**6** and **16**), and to discover differences that could explain their selectivity profiles. It is well-known that the hMAO-B binding site is gated by residue Ile199 and can be longer and more hydrophobic compared to the hMAO-A binding site [39].

**Figure 6:**
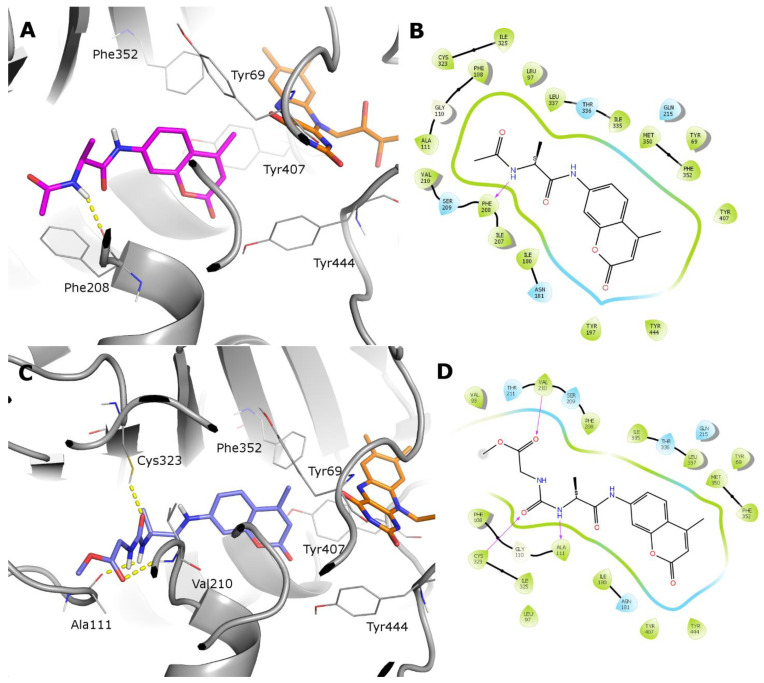
Panels (**A**,**C**) show the catalytic site of hMAO-A (grey cartoon) in complex with compounds **6** (magenta C atoms) and **16** (pale purple C atoms), respectively. Panels (**B**,**D**) show the corresponding 2D interaction diagrams.

The docking poses in the hMAO-A pocket show coumarins **6** and **16** enclosed within the hydrophobic portion produced by the FAD and residues Tyr407, Tyr444, Phe352, and Tyr69. Furthermore, the terminal amide NH of **6** forms an H-bond with the carbonyl oxygen of Phe208, whereas three H-bonds stabilize compound **7**: the urea NH with the carbonyl oxygen of Ala111, the urea C=O with the thiol of Cys32, and the terminal carbonyl with the NH of Val210 (Figure 6).

Since the hydrophobic area of hMAO-B is more extended than in hMAO-A, in hMAO-B coumarins **6** and **16** are bound within the hydrophobic cage formed by FAD, Tyr326, Tyr398, and Tyr435. However, they do not find productive H-bonding interactions or π-π stacking with Tyr398 and Tyr435 as commonly described for coumarin derivatives [7,39,40]. In an attempt to find optimal binding orientations and interactions, the docking algorithm fits the ligands in a reversed orientation compared to their orientations in hMAO-A. Furthermore, for compound **16**, an H-bond is predicted to form between the amine of the urea and the carbonyl oxygen of Leu171 (Figure S7).

The activity profile of studied ligands is affected by the substitution pattern of the coumarin ring: by comparing these results with those already studied and more potent coumarin derivatives [40,41], it is evident that the substitution in 7-position of the 2*H*-chromen-2-one ring is not optimal for hMAO-B inhibition: the most active compounds usually are 3-substituted coumarins with small hydrophobic portions that occupy the longer hydrophobic pocket of hMAO-B, while the coumarin ring is stacked between Tyr398 and Tyr495. Fewer examples of potent 7-substituted ligands are known [42]. The 4,7-substitution pattern appears to be responsible for the relative lower potencies of the study compounds for both isoforms and, in particular, toward MAO-B. This is due to the steric hindrance that occurs when placing the ligand in the correct orientation in the enzyme active site. To further investigate this observation of the role of the substitution pattern, the fit of compounds **6** and **16** in the calculated hydrophobic interaction regions of the SiteMap for both enzyme binding sites are reported in Figure S9. In hMAO-A, both ligands occupy the binding site almost completely with the coumarin ring and its substituent in position four fitting into the hydrophobic site, while the peptide chain bends to reach H-bonding interactions outside the hydrophobic pocket. In hMAO-B, the reversed binding mode of the ligand produces an unfavorable occupation of the binding site by the peptide portion; the coumarin ring is partially embedded in the hydrophobic region with the 4-methyl substituent falling outside the calculated apolar site of the SiteMap.

To explore the possible dual activity of peptidyl coumarins toward both human Cas and MAOs, the Ligand Efficiency was calculated (Table S1). Compound **6** showed the best values for all enzymes. This confirms the validity of the core structure and provides an opportunity to start from this compound for future optimization studies.

To conclude these experiments, an in silico ADME predictive study was conducted (Table S2), which disclosed some issues concerning the drug-likeness profiles of the studied ligands. Their predicted physico-chemical properties are consistent with only moderate oral bioavailability, which may be due to limited cell permeability. On the contrary, the predicted blood-brain distribution is low, which would be detrimental only for hMAO inhibitors that act in the brain [43], and not in other body tissues.

## 4. Conclusions

The potential of linking the coumarin nucleus to a distinctive (*pseudo*)-peptide portion to target tumor-associated hCAs (IX/XII) and the hMAOs have been demonstrated in this study exploring an emerging field of interest of dual modulators [44]. The novel molecules have proven to potently inhibit hCAs IX/XII in vitro, without affecting the I and II cytosolic human isoforms. SAR analysis, in combination with computational studies, provided some interesting insights into structural determinants of the peptide fragment which contribute to the inhibitory profiles and isoform-selectivity of derivatives.

In the two diverging series of compounds, binding affinity for hCA is essentially driven by polar interactions, which mainly involve the peptide backbone functionalities, and secondly the hydroxycinnamic acid moiety, as corroborated by in silico results. Accordingly, in the group of amino acyl coumarins, the smallest and less hydrophobic protecting groups at the amino-terminal position of compounds appear to be beneficial for the inhibition of both isoforms, with alanine being the most optimal size for the residue directly bound to the 7-amino-coumarin core (compounds **6** and **7**). Most interestingly, the urea motif plays a key role in the (*pseudo*)-peptide inhibitors’ selectivity towards the hCA XII isoform. Here molecular docking and MD simulations performed for the most active compound **19** provided a plausible explanation for the observed enzymatic selectivity, which is based on the stability of the peculiar H-bonding network which characterizes the ligand-hCA XII complex. In conclusion, given their prevalent and significant hCA IX and XII inhibitory activities, the study compounds appear to be promising leads for the search for tumor-selective agents. Besides compound **19**, other urea-based peptidyl derivatives can be investigated to gain further insights into how structural changes contribute to isoform-selectivity between the two isoforms.

Furthermore, the novel compounds behave as moderate inhibitors of hMAOs, with selectivity for the MAO-A isoform. While all compounds were found to be weak hMAO-B inhibitors, three compounds (**1**, **6**, and **16**) exhibited moderate hMAO-A inhibition with IC_50_ < 10 µM. The smaller steric size of both the coumarin and (*pseudo*)-peptidyl moieties was favorable for MAO-A inhibition.

In summary, this work resulted in the identification of dual inhibitors of cancer-related hCAs and hMAOs. In particular, compound **6** can be considered a valid starting point for the optimization of activity toward both targets and improving the physicochemical and pharmacokinetic profile of this scaffold.

## 5. Experimental Section

### 5.1. Chemical Synthesis

Solvents and other reagents were purchased from Merck (Milan, Italy) and used as supplied without further purification. Coumarins and amino acids were provided by Iris Biotech GmbH (Marktredwitz, Germany) and Merck (Milan, Italy). Boc-Ala-OH and HCl · H-Leu-OMe were purchased from Merck-Millipore (Milan, Italy). Esters H-β-Ala-OMe, H-β-Ala-OEt, and H-Leu-OMe, commercially available as hydrochlorides, were provided by Bachem (Bubendorf, Switzerland). Mixtures of solvents are specified with the stated ratios as volume/volume. All reactions involving air- or moisture-sensitive compounds were performed under a nitrogen atmosphere using dried glassware and syringe techniques to transfer solutions. Melting points were determined on a Büchi B-450 apparatus and are uncorrected. Chromatographic purifications were performed by Merck 60 (70–230 mesh ASTM) silica gel column. Analytical thin layer chromatography (TLC) was carried out on Merck 60 F_254_ silica gel PET plates. Next, ^1^H-(300 MHz) and ^13^C-(75.43 MHz) NMR spectra were recorded on a 300 MHz Varian VXR spectrometer using a suitable deuterated solvent. Chemical shifts are expressed as parts per million (*δ*) downfield from the internal standard tetramethylsilane (Me_4_Si), and multiplicities are indicated as s (singlet), d (doublet), dd (doublet of doublets), ddd (doublet of doublets of doublets), t (triplet), q (quartet), m (multiplet), and br (broad signal); coupling constants (J) are reported in Hertz (Hz).

#### 5.1.1. General Procedure for the Synthesis of Z/Fmoc-Xaa-AMC/AMMC Derivatives (**1**–**3**, **8**, and **9**)

A stirred solution of *Z/Fmoc-Xaa-OH* (0.97 mmol) in dry THF (3 mL) was kept under nitrogen at −10 °C, and IBCF (0.13 g, 0.97 mmol) and TEA (0.098 g, 0.97 mmol) were added portion-wise over a period of 5 min, followed after 10 min by a solution of AMC/AMMC (0.97 mmol) in dry THF (6 mL). After stirring for 1 h at −10 °C and 20–24 h at room temperature, the reaction mixture was evaporated to dryness under a *vacuum*, the residue taken up in CHCl_3_, and the organic layer washed with 5% KHSO_4_ and H_2_O. The raw materials obtained after solvent drying and evaporation were chromatographed on silica gel, using a gradient of CHCl_3_/MeOH as eluent. Solid products were furtherly purified by crystallization with the opportune solvent(s).

Z-Ala-AMC (**1**)

White solid (32% yield); *R*_f_ (CHCl_3_/MeOH 98:2) = 0.4; m.p. 175–177 °C (MeOH). Chromatography eluent: CHCl_3_/MeOH (98:2). ^1^H-NMR (DMSO-d*_6_*): δ 10.45 (s, 1H, AMC NH), 7.76 (s, 1H, ArH), 7.71 (d, 1H, *J* = 8.3 Hz, ArH), 7.49 (d, 1H, *J* = 8.3 Hz, ArH), 7.38–7.30 (m, 5H, ArH), 7.18 (br, 1H, Ala NH), 6.25 (s, 1H, C=CH), 5.01 (s, 2H, OCH_2_), 4.19 (app t, 1H, Ala α-CH), 2.38 (s, 3H, AMC CH_3_), 1.29 (d, 3H, *J* = 6.6 Hz, Ala CH_3_ ^3^C-NMR (DMSO-d*_6_*): δ 172.9 (CONH), 160.6 (AMC COO), 156.3 (OCONH), 154.1, 153.6, 142.7, 137.3, 128.8, 128.3, 128.2, 126.4, 115.7, 115.5, 112.7, and 106.1 (aromatics), 65.9 (OCH_2_), 51.4 (Ala C^α^), 18.4, and 18.2 (Ala C^β^ and CH_3_).

Fmoc-Ala-AMC (**2**)

White solid (68% yield). *R*_f_ (CHCl_3_/MeOH 98:2) = 0.3; m.p. 153–155 °C (MeOH/AcOEt). Gradient chromatography eluent: CHCl_3_/MeOH (99:1 → 95:5). ^1^H-NMR (DMSO-d*_6_*): δ 10.45 (s, 1H, AMC NH), 7.87 (d, 2H, *J* = 7.0 Hz, ArH), 7.79–7.69 (m, 5H, ArH), 7.54 (d, 1H, *J* = 7.0 Hz, ArH), 7.42–7.29 (m, 4H, ArH and Ala NH), 6.25 (s, 1H, C=CH), 4.28–4.26 (m, 3H, OCH_2_CH), 4.21 (q, 1H, *J* = 7.0 Hz, Ala α-CH), 2.38 (s, 3H, AMC CH_3_), 1.31 (d, 3H, *J* = 7.0 Hz, Ala CH_3_). ^13^C-NMR (DMSO-d*_6_*): δ 173.0 (CONH), 160.9 (AMC COO), 156.3 (OCONH), 154.0, 144.2, 142.6, 141.1, 136.0, 130.0, 128.2, 127.6, 125.6, 124.4, 121.6, 120.5, and 115.6 (aromatics), 69.4 (OCH_2_), 47.00 (Ala C^α^), 18.4, and 18.1 (Ala C^β^ and CH_3_).

Fmoc-Ala-AMMC (**3**)

Pale yellow sticky solid (67% yield). *R*_f_ (CHCl_3_/MeOH 90:10) = 0.7. Gradient chromatography eluent: CHCl_3_/MeOH (97:3 → 90:10). ^1^H-NMR (DMSO-d_6_): δ 10.52 (s, 1H, AMMC NH), 7.86 (d, 2H, *J* = 7.3 Hz, ArH), 7.79 (s, 1H, ArH), 7.77–7.64 (m, 4H, ArH), 7.62 (d, 1H, *J* = 8.8 Hz, ArH), 7.48–7.31 (m, 4H, ArH and Ala NH), 6.28 (s, 1H, C=CH), 4.64 (s, 2H, AMMC OCH_2_), 4.29–4.27 (m, 3H, OCH_2_CH), 4.21 (q, 1H, *J* = 7.0 Hz, Ala α-CH), 3.39 (s, 3H, AMMC OCH_3_), 1.32 (d, 3H, *J* = 7.0 Hz, Ala CH_3_).

Z-Ile-AMMC (**8**)

Pale yellow solid (32% yield). *R*_f_ (CHCl_3_/MeOH (98:2) = 0.6; m.p. 78–80 °C (AcOEt). Chromatography eluent: CHCl_3_/MeOH (99:1). ^1^H-NMR (CDCl_3_): δ 9.26 (s, 1H, AMMC NH), 7.71 (s, 1H, ArH), 7.32–7.26 (m, 6H, ArH), 7.12 (d, 1H, *J* = 8.7 Hz, ArH), 6.35 (s, 1H, C=CH), 5.79 (d, 1H, *J* = 8.7 Hz, Ile NH), 5.12 (ABq, 2H, *J* = 12.3 Hz, OCH_2_), 4.50 (ABq, 2H, *J* = 15.0 Hz, AMMC OCH_2_), 4.26 (app t, 1H, Ile α-CH), 3.49 (s, 3H, OCH_3_), 1.95 (m,1H, Ile β-CH), 1.62 (m, 1H, Ile γ-CH_A_), 1.21 (m, 1H, Ile γ-CH_B_), 0.99 (d, 3H, *J* = 6.6 Hz, Ile γ’-CH_3_), 0.90 (t, 3H, *J* = 7.2 Hz, Ile δ-CH_3_). ^13^C-NMR (CDCl_3_): δ 171.0 (CONH), 161.1 (AMC COO), 157.1 (OCONH), 154.0, 151.6, 141.0, 135.9, 124.2, 115.6, 113.4, 111.2, and 107.3 (aromatics), 70.0 (AMMC OCH_2_), 67.4 (OCH_2_), 60.6 (AMMC OCH_3_), 59.1 (Ile C^α^), 37.0 (Ile C^β^), 24.9 (Ile C^γ^), 15.5 (Ile C^γ^’), 10.9 (Ile C^δ^).

Fmoc-tLeu-AMMC (**9**)

Yellow oil (53% yield). *R*_f_ (CHCl_3_/MeOH 98:2) = 0.4. Chromatography eluent: CHCl_3_/MeOH (98:2). ^1^H-NMR (CDCl_3_): δ 8.94 (s, 1H, AMMC NH), 7.74 (d, 2H, *J* = 6.9 Hz, ArH), 7.56 (d, 2H, *J* = 7.2 Hz, ArH), 7.38 (t, 2H, *J* = 7.2 Hz, ArH), 7.31–7.24 (m, 5H, ArH), 6.39 (s, 1H, C=CH), 5.86 (d, 1H, *J* = 9.0 Hz, *t*Leu NH), 4.58–4.35 (m, 4H, AMMC OCH_2_, OCH_2_C*H*, and *t*Leu α-CH), 4.20 (ABq, 2H, *J* = 9.9 Hz, OC*H_2_*CH), 3.47 (s, 3H, AMMC OCH_3_), 1.08 (s, 9H, *t*Leu CH_3_). ^13^C-NMR (CDCl_3_): δ 170.1 (CONH), 161.1 (AMC COO), 157.0 (OCONH), 154.2, 151.4, 143.6, 142.4, 141.3, 140.8, 127.8, 127.1, 125.0, 124.9, 124.3, 120.0, 115.7, 111.4, and 107.6 (aromatics), 70.1 (AMMC OCH_2_), 67.5 (OCH_2_), 63.7 (AMMC OCH_3_), 59.0 (*t*Leu C^α^), 34.6 (*t*LeuC^β^), 26.5 (*t*Leu C^γ^).

#### 5.1.2. General Protocol for the N-Deprotection of Fmoc-Xaa-AMC/AMMC Derivatives (**2**, **3**, **9**): Preparation of **4**, **5,** and **10**

To a stirred solution of Fmoc-Xaa-AMC/AMMC (4.66 mmol) in DCM (30 mL), DBU (0.71 g, 4.66 mmol) in DCM (4 mL) was added dropwise at room temperature. After 15 min the solution was evaporated to dryness and the residue was chromatographed on silica gel using an appropriate solvent mixture as eluent, to give the pure *N*-deprotected derivative. Spectral characterization is reported only for compound **4**.

H-Ala-AMC (**4**)

Pale yellow solid (83% yield). *R*_f_ (CHCl_3_/MeOH 90:10) = 0.2; m.p. 214–216 °C (MeOH). Gradient chromatography eluent: CHCl_3_/MeOH (95:5 → 85:15). ^1^H-NMR (DMSO-d*_6_*) (amine protons are not observable): δ 10.59 (br, 1H, AMC NH), 8.01 (s, 1H, ArH), 7.70 (d, 1H, *J* = 9.0 Hz, ArH), 7.47 (d, 1H, *J* = 9.0 Hz, ArH), 6.25 (s, 1H, C=CH), 4.51 (q, 1H, *J* = 6.9 Hz, Ala α-CH), 2.38 (s, 3H, AMC CH_3_), 1.30 (d, 3H, *J* = 6.9 Hz, Ala CH_3_). ^13^C-NMR (DMSO-d*_6_*): δ 172.4 (CONH), 160.9 (AMC COO), 153.9, 142.4, 126.4, 118.8, 116.0, 112.6, and 106.2 (aromatics), 51.2 (Ala C^α^), 20.9 (Ala C^β^), 18.4 (CH_3_).

H-Ala-AMMC (**5**)

Pale yellow solid (96% yield). *R*_f_ (CHCl_3_/MeOH 90:10) = 0.3; m.p. 158–160 °C (MeOH). Gradient chromatography eluent: CHCl_3_/MeOH (95:5 → 90:10).

*H-*t*Leu-AMMC (***10***)* Yellow oil (81% yield). Gradient chromatography eluent: CHCl_3_/MeOH (95:5 → 85:15). R*_f_* (CHCl_3_/MeOH 85:15) = 0.15.

#### 5.1.3. General Procedure for the Synthesis of Ac-Ala-AMC/AMMC (**6** and **7**)

The *N*-deprotected derivative (0.81 mmol) was solved in glacial AcOH (5 mL) and the stirred solution was treated with Ac_2_O (0.10 g, 0.97 mmol). After 21 h under stirring at room temperature, the solution was evaporated *in vacuo*, and the resulting material was repeatedly taken up in dry Et_2_O before being chromatographed on a silica gel column using a CHCl_3_/MeOH (90:10) mixture as the eluent.

Ac-Ala-AMC (**6**)

Pale yellow powder (87% yield). *R*_f_ (CHCl_3_/MeOH 90:10) = 0.45; m.p. 237–238 °C (MeOH). ^1^H-NMR (CD_3_OD) (rapidly exchanging amide protons are not observable): δ 7.79 (s, 1H, ArH), 7.68 (d, 1H, *J* = 8.8 Hz, ArH), 7.45 (d, 1H, *J* = 8.8 Hz, ArH), 6.21 (s, 1H, C=CH), 4.46 (q, 1H, *J* = 7.3 Hz, Ala α-CH), 2.43 (s, 3H, AMC CH_3_), 2.01 (s, 3H, COCH_3_), 1.43 (d, 3H, *J* = 7.3 Hz, Ala CH_3_). ^13^C-NMR (CD_3_OD): δ ^13^C-NMR (DMSO-d*_6_*): δ 171.7 (CONH), 170.1 (CONH), 160.1 (AMC COO), 153.3, 152.7, 141.6, 125.0, 114.8, 111.7, 110.0, and 105.5 (aromatics), 49.0 (Ala C^α^), 21.0 (*C*H_3_CO), 16.8 and 16.6 (Ala C^β^ and CH_3_).

Ac-Ala-AMMC (**7**)

Yellow powder (76% yield). *R*_f_ (CHCl_3_/MeOH 90:10) = 0.5; m.p. 208–210 °C (MeOH). ^1^H-NMR (CDCl_3_): δ 9.96 (s, 1H, AMMC NH), 7.79 (s, 1H, ArH), 7.38 (d, 1H, *J* = 8.7 Hz, ArH), 7.27 (d, 1H, *J* = 8.7 Hz, ArH), 7.26 (d, 1H, *J* = 7.5 Hz, Ala NH), 6.41 (s, 1H, C=CH), 4.78 (app t, 1H, Ala α-CH), 4.56 (s, 2H, AMMC OCH_2_), 3.44 (s, 3H, AMMC OCH_3_), 2.01 (s, 3H, CH_3_CO), 1.49 (d, 3H, *J* = 7.5 Hz, Ala CH_3_). ^13^C-NMR (CDCl_3_): δ 171.9 (CONH), 171.3 (CONH), 161.2 (AMC COO), 154.1, 151.6, 141.5, 124.2, 120.1, 115.8, 111.1, and 107.4 (aromatics), 70.0 (AMMC OCH_2_), 59.0 (AMMC OCH_3_), 50.2 (Ala C^α^), 23.1 (*C*H_3_CO), 18.1 (Ala C^β^).

#### 5.1.4. Synthesis of Z-Ala-tLeu-AMMC (**11**)

To a stirred solution of Z-Ala-OH (0.105 g, 0.47 mmol) in dry THF (2 mL), kept under nitrogen at −10 °C, IBCF (0.064 g, 0.47 mmol) and TEA (0.048 g, 0.47 mmol) were added portion-wise over a period of 5 min, followed after 10 min by a solution of **10** (0.149 g, 0.47 mmol) in dry THF (3 mL). After stirring for 1 h at −10 °C and 24 h at r.t., the reaction mixture was evaporated to dryness *in vacuo*, and the residue was taken up in CHCl_3_. The organic layer was washed with 5% KHSO_4_ and H_2_O, dried, and evaporated under reduced pressure to furnish a raw material, which was eluted from a silica gel column using CHCl_3_/MeOH (98:2) as the eluent, to give compound **11** as a pure white solid (0.11 g, 45%). *R*_f_ (CHCl_3_/MeOH 98:2) = 0.2; m.p. 205–209 °C (MeOH/AcOEt). ^1^H-NMR (CDCl_3_): *δ* 9.20 (s, 1H, AMMC NH), 7.71 (s, 1H, ArH), 7.42–7.25 (m, 8H, ArH and *t*Leu NH), 6.39 (s, 1H, C=CH), 5.79 (br, 1H, Ala NH), 5.15 (s, 2H, OCH_2_), 4.55–4.47 (m, 4H, AMMC OCH_2_, *t*Leu and Ala α-CH), 3.48 (s, 3H, AMMC OCH_3_), 1.40 (d, 3H, *J* = 7.2 Hz, Ala CH_3_), 1.04 (s, 9H, *t*Leu CH_3_). ^13^C-NMR (CDCl_3_): δ 173.0 (CONH), 169.5 (CONH), 161.0 (AMC COO), 156.3 (OCONH), 154.2, 151.3, 141.0, 136.0, 128.5, 128.2, 128.0, 124.2, 116.0, 113.7, 111.4, and 107.7 (aromatics), 70.1 (AMMC OCH_2_), 67.2 (OCH_2_), 61.8 (AMMC OCH_3_), 59.0 (*t*Leu C^α^), 50.8 (Ala C^α^), 34.7 (*t*Leu C^β^), 26.6 (*t*Leu C^γ^), 18.2 (Ala C^β^).

#### 5.1.5. General Protocol for the Preparation of Active Carbamates (**12**–**15**)

A stirred solution of H-Xaa-OMe/OEt.HCl (1.33 mmol) in dry DCM (2 mL) was treated with Py (0.11 g, 1.39 mmol) at room temperature, and a solution of *p*-NO_2_-phenyl cloroformate (0.28 g, 1.39 mmol) in dry DCM (1 mL) was added. The reaction mixture was kept under stirring for 24 h, then evaporated under reduced pressure, and the residue was taken up in CHCl_3_. The solution was repeatedly and successively washed with 5% KHSO_4_, Na_2_CO_3_ (saturated), and H_2_O. The residue obtained after drying and evaporation of the solvent was eluted from a silica gel column using CHCl_3_/hexane (97:3) as eluent, to give the expected carbamate.

p-NO_2_-Ph-OCO-Gly-OMe (**12**)

White foam (yield 34%). *R*_f_ (CHCl_3_/hexane 97:3) = 0.1. ^1^H-NMR (CDCl_3_): δ 8.25 (d, 2H, *J* = 6.6 Hz, ArH), 7.33 (d, *J* = 6.6 Hz, 2H, ArH), 5.71 (br t, 1H, Gly NH), 4.1 (d, 2H, *J* = 5.7 Hz, Gly α-CH_2_), 3.81 (s, 3H, COOCH_3_).

p-NO_2_-Ph-OCO-Ala-OMe (**13**)

White foam (yield 70%). *R*_f_ (CHCl_3_/hexane 97:3) = 0.6. ^1^H-NMR (CDCl_3_): δ 8.23 (d, 2H, *J* = 8.7 Hz, ArH), 7.32 (d, 2H, *J* = 8.7 Hz, ArH), 5.79 (br d, 1H, Ala NH), 4.47 (app t, 1H, Ala α-CH), 3.79 (s, 3H, COOCH_3_), 1.50 (d, 3H, *J* = 7.2 Hz, Ala CH_3_).

p-NO_2_-Ph-OCO-Phe-OMe (**14**)

White foam (yield 26%). *R*_f_ (CHCl_3_/hexane 97:3) = 0.15). ^1^H-NMR (CDCl_3_): δ 8.22 (d, 2H, *J* = 7.2 Hz, ArH), 7.33 (d, 2H, *J* = 7.2 Hz, ArH), 7.29–7.25 (m, 3H, ArH), 7.18 (d, 2H, *J* = 7.5 Hz, ArH), 5.64 (d, 1H, *J* = 8.1 Hz, Phe NH), 4.7 (ddd, 1H, *J* = 6.0 Hz, *J* = 8.1 Hz, *J* = 13.8 Hz, Phe α-CH), 3.79 (s, 3H, COOCH_3_), 3.2 (ddd, 2H, *J* = 6.0 Hz, *J* = 13.8 Hz, *J* = 29.1 Hz, Phe β-CH_2_).

p-NO_2_-Ph-OCO-βAla-OEt (**15**)

Pale yellow wax (yield 29%). *R*_f_ (CHCl_3_/hexane 97:3) = 0.3. ^1^H-NMR (CDCl_3_): δ 8.22 (d, 2H, *J* = 8.9 Hz, ArH), 7.29 (d, 2H, *J* = 8.9 Hz, ArH), 5.80 (br t, 1H, βAla NH), 4.18 (q, 2H, *J* = 7.0 Hz, O*CH_2_*CH_3_), 3.55 (dd, 2H, *J* = 6.2 Hz, *J* = 11.9 Hz, βAla β-CH_2_), 2.61 (dd, 2H, *J* = 6.2 Hz, *J* = 11.9 Hz, βAla α-CH_2_), 1.28 (t, 3H, *J* = 7.0 Hz, OCH_2_C*H_3_*).

#### 5.1.6. General Procedure for the Synthesis of (*Pseudo*)-Dipeptidyl Coumarins (**16**–**20**)

To a stirred solution of H-Ala-AMC/AMMC (**6**/**7**) (1.27 mmol) in dry DMF (10 mL) and catalytic amounts of DMAP (0.039 g, 0.32 mmol) a solution of carbamate (**12**–**15**) (1.27 mmol) in dry DMF (5 mL) was added portion-wise at room temperature. After stirring for 72 h at room temperature, the reaction mixture was evaporated under a vacuum and the residue was taken up in EtOAc. The organic layer was washed with 5% KHSO_4_, Na_2_CO_3_ (saturated), and H_2_O, dried, and evaporated under reduced pressure to furnish the expected compound as raw material. Purification by silica gel column chromatography of the resulting crude product, using CHCl_3_/MeOH (95:5) as the eluent, afforded the expected pure urea derivatives.

MeO-Gly-CO-Ala-AMC (**16**)

White solid (yield 44%). *R*_f_ (CHCl_3_/MeOH 95:5) = 0.15; m.p. 193–195 °C (MeOH). ^1^H-NMR (DMSO-*d_6_*): δ 10.46 (s, 1H, AMC NH), 7.76 (d, 1H, *J* = 2.0 Hz, ArH), 7.69 (d, 1H, *J* = 8.7 Hz, ArH), 7.45 (dd, 1H, *J* = 8.7 Hz, *J* = 2.0 Hz, ArH), 6.65 (d, 1H, *J* = 7.7 Hz, Ala NH) 6.41 (t, 1H, *J* = 6.2 Hz, Gly NH), 6.24 (s, 1H, C=CH), 4.32 (app t, 1H, Ala α-CH), 3.78 (d, 2H, *J* = 6.2 Hz, Gly α-CH_2_), 3.59 (s, 3H, COOCH_3_), 2.37 (s, 3H, MAC CH_3_), 1.26 (d, 3H, *J* = 6.7 Hz, Ala CH_3_). ^13^C-NMR (DMSO-d*_6_*): δ 173.5 (CONH), 172.0 (COO), 160.6 (AMC COO), 157.8 (NHCONH), 154.0, 153.7, 142.7, 126.4, 115.7, 115.5, 112.7, and 106.1 (aromatics), 52.0 (OCH_3_), 50.1 (Ala C^α^), 41.7 (Gly C^α^), 19.4, and 18.4 (Ala C^β^ and CH_3_).

MeO-Ala-CO-Ala-AMC (**17**)

White solid (yield 80%). *R*_f_ (CHCl_3_/MeOH 95:5) = 0.4; m.p. 193–195 °C (MeOH). ^1^H-NMR (DMSO-*d*_6_): δ 10.45 (s, 1H, AMC NH), 7.76 (s, 1H, ArH), 7.71 (d, 1H, *J* = 8.7 Hz, ArH), 7.48 (d, 1H, *J* = 8.7 Hz, ArH), 6.48 (d, 1H, *J* = 7.8 Hz, Ala NH), 6.41 (d, 1H, *J* = 7.5 Hz, Ala NH), 6.25 (s, 1H, C=CH), 4.28 (m, 1H, Ala α-CH), 4.13 (m, 1H, Ala α-CH), 3.60 (s, 3H, COOCH_3_), 2.38 (s, 3H, AMC CH_3_), 1.24 (d, 3H, *J* = 7.2 Hz, Ala CH_3_), 1.23 (d, 3H, *J* = 7.2 Hz, Ala CH_3_). ^13^C-NMR (DMSO-d*_6_*): δ 173.5 (CONH), 173.4 (COO), 160.4 (AMC COO), 157.2 (NHCONH), 154.1, 153.5, 142.7, 126.4, 126.3, 115.6, 115.4, 112.7, and 106.0 (aromatics), 52.1 (OCH_3_), 50.0 (Ala C^α^), 48.5 (Ala C^α^), 19.4, and 18.4 (Ala C^β^ and CH_3_).

MeO-Phe-CO-Ala-AMC (**18**)

Pale orange solid (yield 91%). *R*_f_ (CHCl_3_/MeOH 95:5) = 0.35; m.p. 177–183 °C (MeOH). ^1^H-NMR (DMSO-d*_6_*): δ 10.45 (s, 1H, AMC NH), 7.75 (d, 1H, *J* = 1.2 Hz, ArH), 7.70 (d, 1H, *J* = 8.7 Hz, ArH), 7.45 (dd, 1H, *J* = 8.7 Hz, *J* = 1.2 Hz, ArH), 7.29–7.14 (m, 5H, ArH), 6.58 (d, 1H, *J* = 7.8 Hz, Ala or Phe NH), 6.41 (d, 1H, *J* = 7.8 Hz, Phe or Ala NH), 6.25 (s, 1H, C=CH), 4.41–4.24 (m, 2H, Ala and Phe α-CH), 3.57 (s, 3H, COOCH_3_), 2.98–2.82 (m, 2H, Phe β-CH_2_), 2.37 (s, 3H, AMC CH_3_), 1.24 (d, 3H, *J* = 6.9 Hz, Ala CH_3_). ^13^C-NMR (DMSO-d*_6_*): δ 173.4 (CONH), 173.2 (COO), 160.6 (AMC COO), 157.3 (NHCONH), 154.0, 153.7, 142.7, 142.5, 137.2, 130.2, 129.6, 128.8, 127.1, 126.4, 115.7, 115.5, 112.7, and 106.1 (aromatics), 54.5 (Phe C^α^), 50.2 and 50.1 (OCH_3_ and Ala C^α^), 37.8 (Phe C^β^), 19.3, and 18.4 (Ala C^β^ and CH_3_).

EtO-βAla-CO-Ala-AMC (**19**)

White solid (yield 66%). *R*_f_ (CHCl_3_/MeOH 95:5) = 0.45. m.p. 182–183 °C (MeOH/AcOEt). ^1^H-NMR (CDCl_3_): δ 10.08 (s, 1H, AMC NH), 7.74 (s, 1H, ArH), 7.44 (d, 1H, *J* = 8.6 Hz, ArH), 7.3 (d, 1H, *J* = 8.6 Hz, ArH), 6.26 (br d, 1H, Ala NH), 6.10 (s, 1H, C=CH), 5.93 (br t, 1H, βAla NH), 4.61 (m, 1H, Ala α-CH), 4.10 (q, 2H, *J* = 7.1 Hz, O*CH_2_*CH_3_), 3.52–3.38 (m, 2H, βAla β-CH_2_), 2.49 (app t, 2H, βAla α-CH_2_), 2.38 (s, 3H, AMC CH_3_), 1.45 (d, 3H, *J* = 7.2 Hz, Ala CH_3_), 1.22 (t, 3H, *J* = 7.1 Hz, OCH_2_*CH_3_*). ^13^C-NMR (CDCl_3_): δ 173.8 (CONH), 172.5 (COO), 161.1 (AMC COO), 158.4 (NHCONH), 153.9, 152.6, 141.7, 125.0, 115.9, 115.7, 113.0, and 107.3 (aromatics), 60.7 (OCH_2_), 50.8 (Ala C^α^), 35.9 (βAla C^α^), 34.8 (βAla C^β^), 18.5, and 18.3 (Ala C^β^ and CH_3_), 14.1 (CH_3_).

MeO-Ala-CO-Ala-AMMC (**20**)

White solid (yield 54%). *R*_f_ (CHCl_3_/MeOH 95:5) = 0.3; m.p. 148–150 °C (MeOH). ^1^H-NMR (DMSO-*d*_6_): δ 10.43 (s, 1H, AMMC NH), 7.76 (s, 1H, ArH), 7.61 (d, 1H, *J* = 9.0 Hz, ArH), 7.42 (d, 1H, *J* = 9.0 Hz, ArH), 6.45 (d, 1H, *J* = 7.5 Hz, Ala NH), 6.37 (d, 1H, *J* = 7.5 Hz, Ala NH), 6.26 (s, 1H, C=CH), 4.62 (s, 2H, AMMC OCH_2_), 4.26 (m, 1H, Ala α-CH), 4.10 (m, 1H, Ala α-CH), 3.57 (s, 3H, COOCH_3_), 3.37 (s, 3H, AMMC OCH_3_), 1.27 (d, 3H, *J* = 7.2 Hz, Ala CH_3_), 1.22 (d, 3H, *J* = 7.2 Hz, Ala CH_3_). ^13^C-NMR (DMSO-d_6_): δ 174.5 (CONH), 173.5 (COO), 161.0 (AMC COO), 157.2 (NHCONH), 152.7, 142.7, 126.6, 125.7, 115.7, 113.0, 110.4, and 106.2 (aromatics), 69.7 (AMMC OCH_2_), 58.8 (AMMC OCH_3_), 52.2 (OCH_3_), 50.0 (Ala C^α^), 48.5 (Ala C^α^), 19.4, and 18.4 (Ala C^β^).

### 5.2. Biological Methods

#### 5.2.1. hCA Inhibition Assay

An Applied Photophysics stopped-flow instrument was used for measuring hCA-catalyzed CO_2_ hydration activity [45]. Phenol red (at a concentration of 0.2 mM) was used as an indicator, the reactions were monitored at a wavelength of 557 nm, and 20 mM Hepes (pH 7.4) containing 20 mM Na_2_SO_4_ (for maintaining constant ionic strength) was used as a buffer. The initial rates of the CA-catalyzed CO_2_ hydration reaction were measured for a period of 10–100 s. The CO_2_ concentrations ranged from 1.7 to 17 mM for the determination of the kinetic parameters and inhibition constants. The non-catalyzed CO_2_ hydration was not subtracted from these curves and accounted for the remaining observed activity, which, even at a high concentration of inhibitors, was in the range of 16–25%. However, the background activity from the uncatalyzed reaction was always subtracted when IC_50_ values were obtained by using the data analysis software for the stopped-flow instrument. Enzyme concentrations ranged between 5 nM and 12 nM. For each inhibitor, at least six graphs of the initial 5–10% of the reaction were used to determine the initial velocity. The uncatalyzed rates were measured in the same manner and were subtracted from the total observed rates. Stock solutions of the inhibitor (0.1 mM) were prepared in distilled-deionized water, and dilutions up to 0.01 nM were performed thereafter with the assay buffer. Inhibitor and enzyme solutions were preincubated together for 15 min at room temperature prior to the assay, to allow for the formation of the E–I complex. The inhibition constants (*K*_i_) were obtained by the nonlinear least-squares methods using PRISM 3 and the Cheng-Prusoff equation as reported earlier and are presented as the mean from at least three different determinations. All hCA isoforms were recombinant proteins obtained in-house, as reported earlier [46].

#### 5.2.2. hMAO Inhibition Studies

Activity measurements (IC_50_) for hMAO-A and hMAO-B were carried out as reported in the literature [47,48]. Recombinant human MAO-A and MAO-B were obtained commercially (Sigma-Aldrich, Cape Town, South Africa) and were used as enzyme sources. Kynuramine is a non-specific substrate and serves as a substrate for both hMAO isoforms. The hMAOs metabolize kynuramine to produce 4-hydroxyquinoline, which was measured at the endpoint of the enzyme reactions by fluorescence spectrophotometry.

### 5.3. Computational Studies

#### 5.3.1. Ligand Preparation

All molecular modeling experiments were performed on Schrödinger Life-Sciences Suite 2021-4 [49]. Ligand structures were built in Maestro using the 2D sketcher. For the hCA docking studies, the hydrolyzed forms of both in *E* and *Z* configurations were also produced. The ligand 3D geometry was obtained using LigPrep to find all possible tautomers and protonation states at pH 7.0 ± 0.4. The obtained structures were energy-minimized with MacroModel, using the OPLS4 force field, and applying 5000 steps of PRCG algorithm with a convergence criterion of 0.001 KJ/molÅ.

#### 5.3.2. hCA Structure Preparation and Docking

The 3D structures of the studied hCAs were retrieved from the Protein Data Bank; protein structures with PDB ID 6F3B for hCA I, 5BNL for hCA II, 6G9U for hCA IX and 5LL5 for hCA XII (resolution 1.40, 2.00, 1.75, and 1.42, respectively) were selected for the structure-based studies. The proteins were prepared using the Protein Preparation routine in Maestro [50] which prepares the protein structure by adding hydrogen atoms, removing water molecules, adjusting the protonation states of ionizable groups, optimizing the H-bond network, and performing a constrained minimization of the final structure. All proteins were aligned to each other using the Protein Structure Alignment module in Maestro. A water molecule that coordinates with the zinc ion was copied from the 5BNL structure to the other hCAs as the fourth zinc ligand to complete its coordination sphere.

Docking calculations were carried out using Glide [51,52]. The Glide Grid suitable for the SP-peptide docking protocol was generated and the center of the grid was set on the X-ray ligand of 5BNL [(2*E*)-3-(2-hydroxy-phenyl)acrylic acid] for all receptors. The grid box dimension of up to 25 Å was set for three ligands. The Glide SP-peptide docking calculation was carried out saving 100 poses for each ligand. Following a previously applied procedure, ligand poses were clustered, and the highest-scored binding geometry of the most populated cluster was further analyzed for each ligand [29,30]. The Multiple Sequence Alignment tool was exploited to measure the sequence identity and similarity among studied hCAs.

#### 5.3.3. Molecular Dynamics of the CA XII:Ligand **19** Complex

The docked complex of compound **19** in the hCA XII protein was submitted to Molecular Dynamics simulation using Desmond [53]. The complex was embedded in an orthorombic box of TIP4P water molecules. The system was neutralized by adding 8 Na^+^ ions.

The solvated complex was submitted to six relaxation steps of 20 ns that preceded the production phase by default. The 100 ns simulation was then carried out recording frames every 100 ps using a normal pressure-temperature (NPT) ensemble with a Nosé-Hoover thermostat at 300 K and Martyna-Tobias-Klein barostat at 1.01325 bar pressure. The Smooth Particle Mesh Ewald method was also applied to analyze the electrostatic interactions with a cut-off distance set to 9.0 Å. The trajectory was analyzed using the Simulation interaction diagram facility in Maestro.

#### 5.3.4. hMAO-A and hMAO-B Protein Structure Preparation and Docking

The X-ray structures of hMAO-A and hMAO-B with PDB ID 2Z5X and 2V5Z were retrieved from the Protein Data Bank (resolution 2.20 Å and 1.60 Å, respectively).

The crystal structures were corrected and minimized using the Protein Preparation wizard in Maestro as already described. Water molecules not directly linked to FAD were removed. Molecular docking analyses were performed using Glide [46]. The Glide grid box was generated by using the center of mass of crystallographic ligands. The following rotatable OH and SH bonds were set: Cys323, Tyr407, Tyr444 for hMAO-A, and Cys172, Tyr398, Tyr435, for MAO-B. The SP docking protocol was applied with the OPLS4 force field. The reliability of the docking protocol was assessed by docking the crystallographic ligands. The Root Mean Square Deviation (RMSD), based on the maximum common structure, of the best-docked poses from the position of the crystallographic ligands were 0.1363 Å, and 0.2279 Å for hMAO-A (2Z5X) and hMAO-B (2V5Z), respectively. SiteMap calculations [54,55] were carried out on the hMAO-A and hMAO-B binding sites to analyze the binding site, and to calculate a fine grid.

## Figures and Tables

**Table 1:** Inhibitory activities, reported as *K*_i_ (nM) values ^a^, of amino acyl-(compounds **1**, **2**, **6**–**8**) and (*pseudo*)-dipeptidyl coumarins (**11**, **16**–**20**) towards hCA I, hCA II, hCA IX, and hCA XII, and corresponding selectivities among the selected hCA isoforms.

Compound	*K*_i_ hCA I	*K*_i_ hCA II	*K*_i_ hCA IX	*K*_i_ hCA XII	SI hCA IX over hCA I or hCA II ^b^	SI hCA XII over hCA I or hCA II ^b^	SI hCA XII over hCA IX ^c^
Z-Ala-AMC (**1**)	>10,000	>10,000	30.5	110.0 ^d^	>327.9	>90.9	0.3
Fmoc-Ala-AMC (**2**)	>10,000	>10,000	91.0	37.2	>109.9	>268.8	2.4
Ac-Ala-AMC (**6**)	>10,000	>10,000	23.4	30.5	>427.3	>327.9	0.8
Ac-Ala-AMMC (**7**)	>10,000	>10,000	29.4	46.8	>340.1	>213.7	0.6
Z-Ile-AMMC (**8**)	>10,000	>10,000	78.7	41.5	>127.1	>241.0	1.9
Z-Ala-*t*Leu-AMMC (**11**)	>10,000	>10,000	171.5	336.3	>58.3	>29.7	0.5
MeO-Gly-CO-Ala-AMC (**16**)	>10,000	>10,000	183.3	38.2	>54.6	>261.8	4.8
MeO-Ala-CO-Ala-AMC (**17**)	>10,000	>10,000	163.3	9.6	>61.2	>1041.7	17.0
MeO-Phe-CO-Ala-AMC (**18**)	>10,000	>10,000	93.6	40.0	>106.8	>250.0	2.3
EtO-βAla-CO-Ala-AMC (**19**)	>10,000	>10,000	260.5	9.5	>38.4	>1052.6	27.4
MeO-Ala-CO-Ala-AMMC (**20**)	>10,000	>10,000	27.0	54.7	>370.4	>182.8	0.5
AAZ	250.0	12.1	25.8	5.7	9.7 0.5	43.8 2.1	4.5

^a^ Data are the mean of three independent experiments, conducted by a stopped-flow technique (errors were in the range of ±5–10% of the reported values). ^b^ SI hCA IX or XII = *K*_i_ hCA I or II (nM)/*K*_i_ hCA IX or XII (nM). ^c^ SI hCA XII *= K*_i_ hCA IX (nM)/ *K*_i_ hCA XII (nM). ^d^ Ref. [16].

**Table 2:** Inhibitory activities reported as IC_50_ μM values ^a^ of amino acyl (compounds **1**, **2**, **6**–**8**) and (*pseudo*)-dipeptidyl coumarins **11**, **16**–**20** against hMAO-A and hMAO-B.

Compound	MAO-A (IC_50_ μM)	MAO-B (IC_50_ μM)
Z-Ala-MAC (**1**)	9.31 ± 0.132	>100
Fmoc-Ala-MAC (**2**)	54.8 ± 0.028	>100
Ac-Ala-MAC (**6**)	1.92 ± 0.247	>100
Ac-Ala-MMAC (**7**)	12.0 ± 0.693	45.6 ± 1.85
Z-Ile-MMAC (**8**)	47.0 ± 13.5	>100
Z-Ala-*t*Leu-MMAC (**11**)	41.3 ± 17.8	79.5 ± 7.24
MeO-Gly-CO-Ala-MAC (**16**)	5.910 ± 0.155	>100
MeO-Ala-CO-Ala-MAC (**17**)	69.6 ± 9.28	58.6 ± 24.0
MeO-Phe-CO-Ala-MAC (**18**)	21.8 ± 0.092	78.0 ± 22.3
EtO-βAla-CO-Ala-MAC (**19**)	>100	>100
MeO-Ala-CO-Ala-MMAC (**20**)	>100	75.3 ± 0.976
Harmine	0.0041 ± 0.00007	-
Isatin	8.43 ± 0.245	3.90 ± 0.792

^a^ Data are given as the mean ± SD of three independent experiments.

## Data Availability

Data are contained within the article.

## Supplementary Materials

The following supporting information can be downloaded at: https://www.mdpi.com/article/10.3390/molecules27227884/s1, Figure S1: Alignment of 3F8E and 5BNL CA II X-ray complexes, and docked posed of compound **19** in CA XII and compound **6** in CA IX; Figure S2: Ligand interaction diagrams; Figure S3: Ligand RMSF; Figure S4: Protein RMSF; Figure S5: Timeline representation of ligand-protein contacts; Figure S6: Depiction of frequency and type of ligand-protein interaction along with the MD simulation; Figure S7: Alignment of CA XII:**19** representative binding geometries obtained from clustering the MD frames; Figure S8: Docked poses of ligands **6** and **16** in MAO-B binding site; Figure S9: SiteMap hydrophobic sites in MAO-A and MAO-B binding site; Table S1: Ligand efficiency values calculated from the inhibition data of studied coumarin derivatives against CAs and MAOs; Table S2: Physico-chemical and pharmacokinetic properties of studied ligands calculated by QuikProp.

## Sample Availability

Samples of all compounds are available from the authors.
