# Supplementary material for: Coumarin-Based Dual Inhibitors of Human Carbonic Anhydrases and Monoamine Oxidases Featuring Amino Acyl and (Pseudo)-Dipeptidyl Appendages: In Vitro and Computational Studies
Source: Molecules. 2022 Nov 15;27(22):7884. doi: 10.3390/molecules27227884 (PMC9692511; doi:10.3390/molecules27227884)
Supplement: Supplementary file 1 [file molecules-27-07884-s001.zip › molecules-2005767-supplementary.pdf]

Supplementary material

Coumarin-Based Dual Inhibitors of Human Carbonic Anhydrases and Monoamine Oxidases Featuring Amino Acyl and (Pseudo)-Dipeptidyl Appendages: In Vitro and Computational Studies

Mariangela Agamennone [1], Marialuigia Fantacuzzi [1], Simone Carradori [1,*], Anél Petzer [2], Jacobus P. Petzer [2], Andrea Angeli [3], Claudiu T. Supuran [3] and Grazia Luisi [1]

[1]*Department of Pharmacy, "G. d'Annunzio" University of Chieti-Pescara, 66100 Chieti, Itay*

[2]*Pharmaceutical Chemistry, School of Pharmacy and Centre of Excellence for Pharmaceutical Sciences, North-West University, Potchefstroom 2520, South Africa*

[3]*Neurofarba Department, Section of Pharmaceutical and Nutraceutical Sciences, University of Florence, Sesto Fiorentino, 50019 Florence, Italy*

* Correspondence: simone.carradori@unich.it

Table of contents

**Figure S1**	Alignment of 3F8E and 5BNL CA II X-ray complexes, and docked posed of compound **19** in CA XII and compound **6** in CA IX	S2
**Figure S2**	Ligand interaction diagrams	S3
**Figure S3**	Ligand RMSF	S4
**Figure S4**	Protein RMSF	S4
**Figure S5**	Timeline representation of ligand-protein contacts	S5
**Figure S6**	Depiction of frequency and type of ligand-protein interaction along with the MD simulation	S5
**Figure S7**	Alignment of CA XII:**19** representative binding geometries obtained from clustering the MD frames.	S6
**Figure S8**	Docked poses of ligands **6** and **16** in MAO-B binding site	S7
**Figure S9**	SiteMap hydrophobic sites in MAO-A and MAO-B binding site	S8
**Table S1**	Ligand efficiency values calculated from the inhibition data of studied coumarin derivatives against CAs and MAOs.	S9
**Table S2**	Physico-chemical and pharmacokinetic properties of studied ligands calculated by QuikProp.	S10

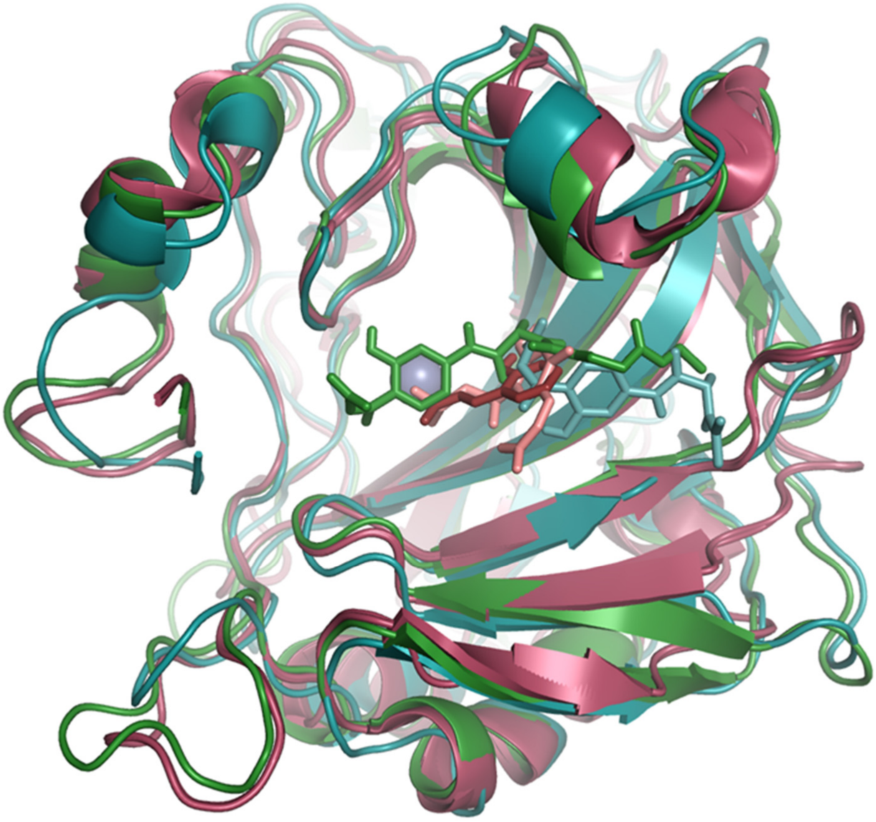

Figure S1. Superposition between 3F8E (salmon) and 5BNL (raspberry) CA II X-ray complexes, and docked poses of compound **6** in the CA IX (cyan) and compound **19** in the CA XII (dark green) proteins. Ligands occupy almost the same region of the enzymes' active site.

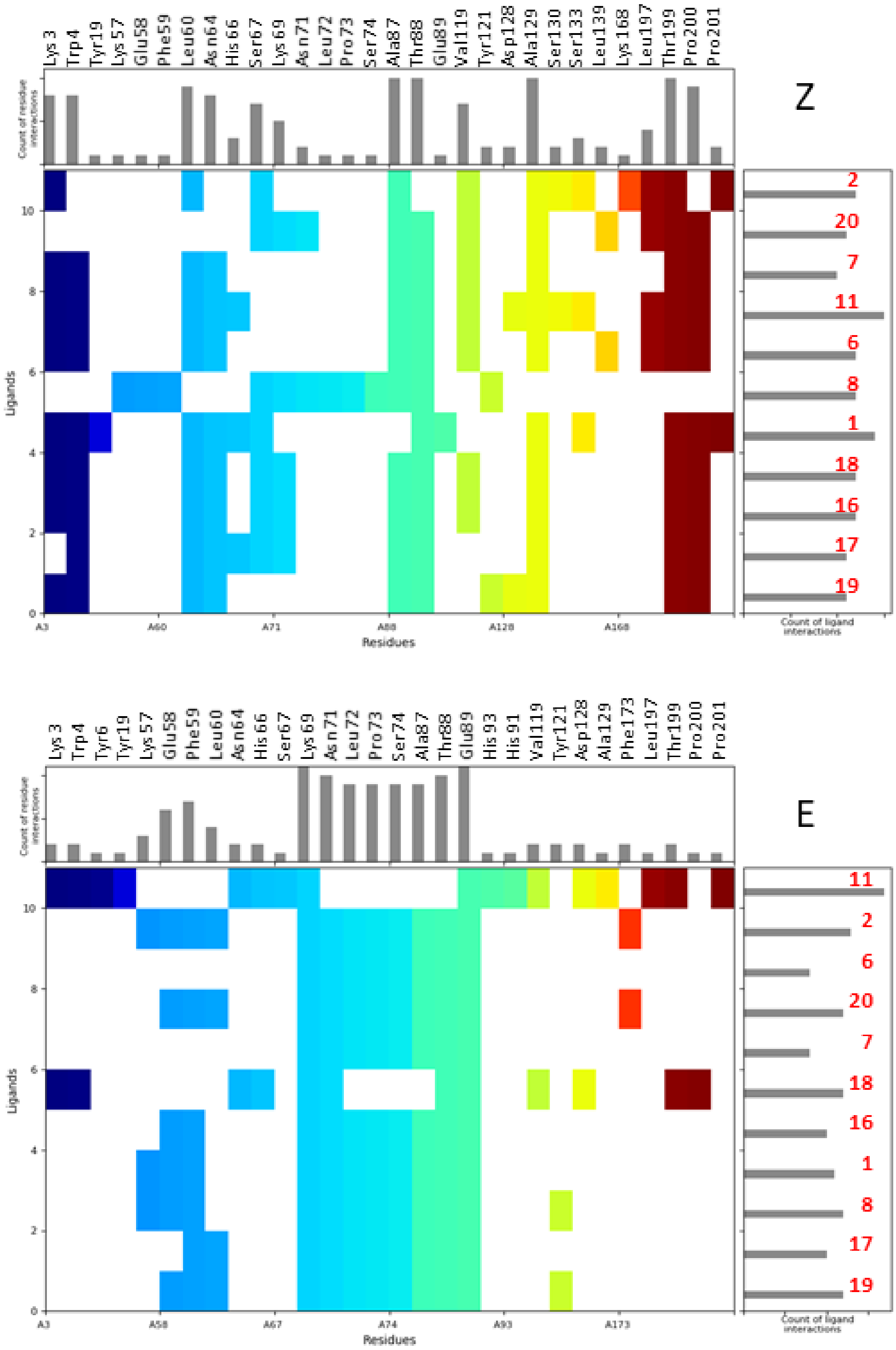

Figure S2. Ligand interaction diagrams for all *Z* and *E* hydrolyzed forms of all studied ligands in the CA XII. The most conserved contacts are shared among all inhibitors.

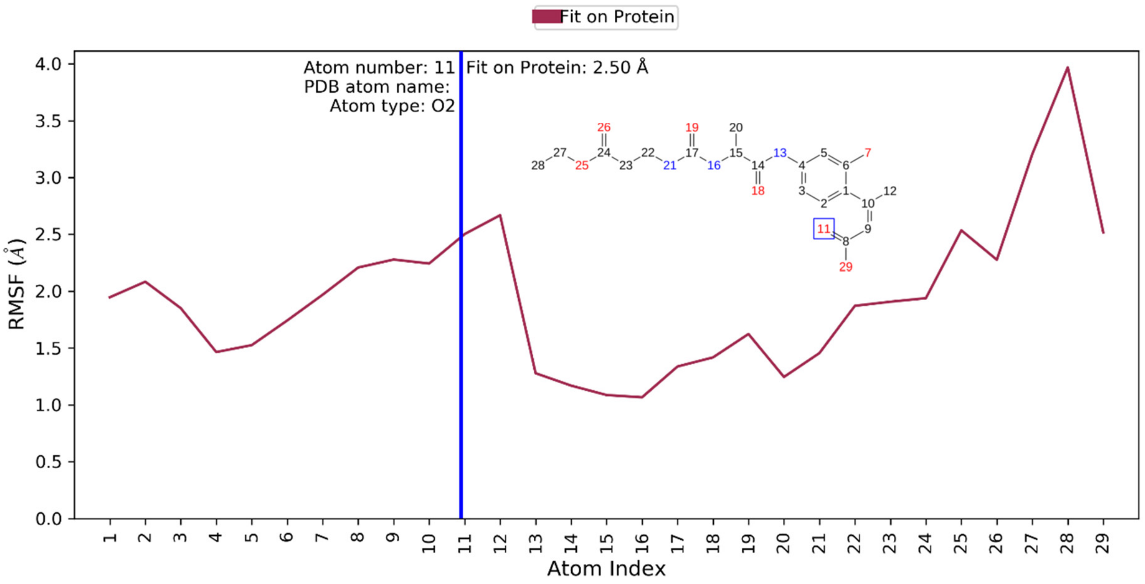

Figure S3. Ligand **19** Root Mean Square Fluctuation (RMSF) calculated in the MD simulation.

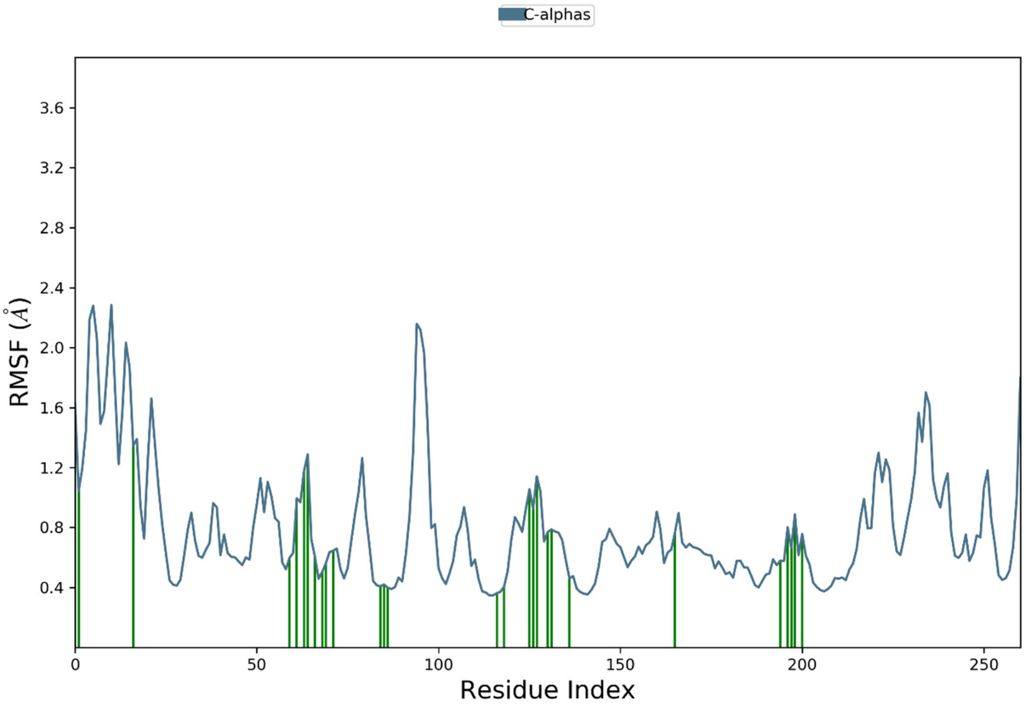

Figure S4. Protein RMSF; green lines indicate residues involved in ligand **19** interactions.

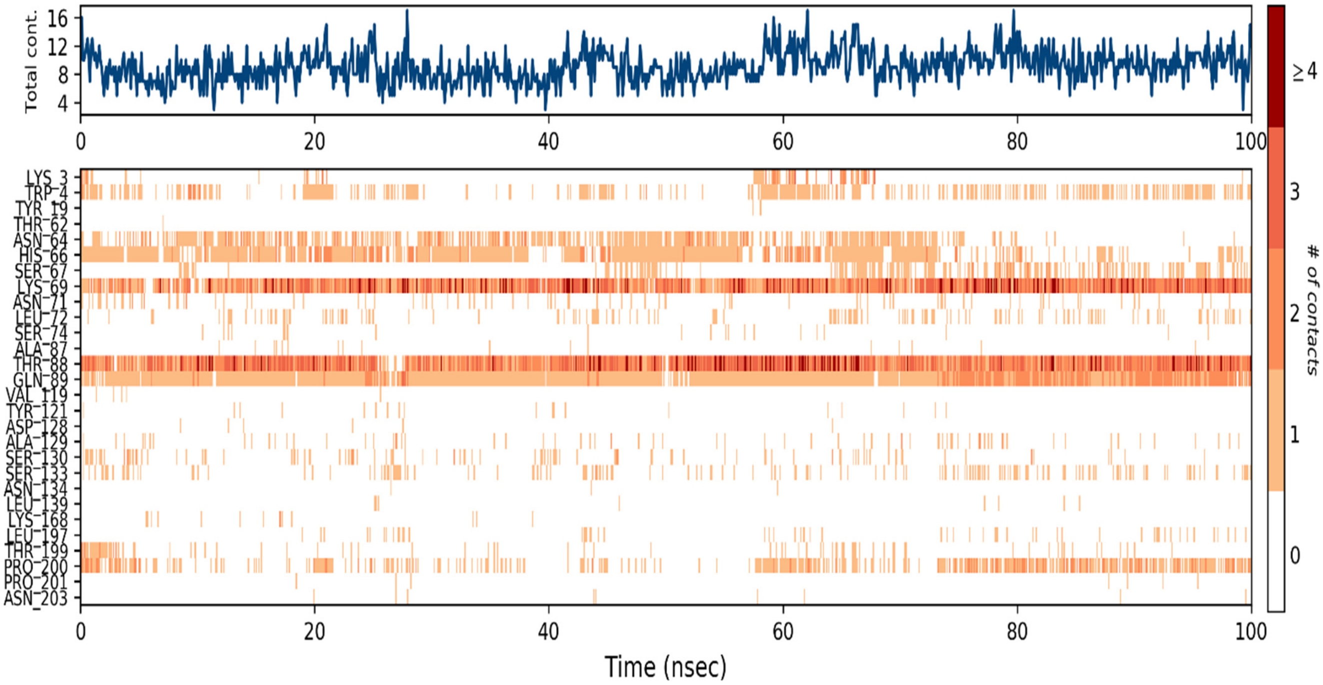

Figure S5. Timeline representation of ligand-protein contacts during MD simulation.

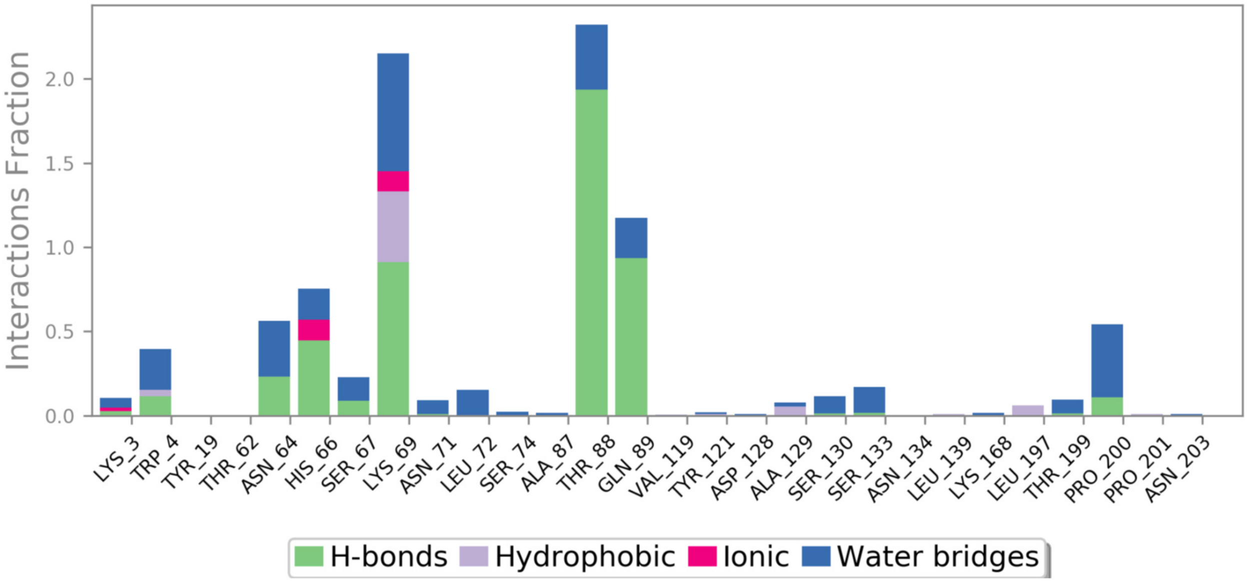

Figure S6. Depiction of frequency and type of ligand-protein interaction along with the MD simulation. Ligand **19** forms mainly polar contacts with the protein.

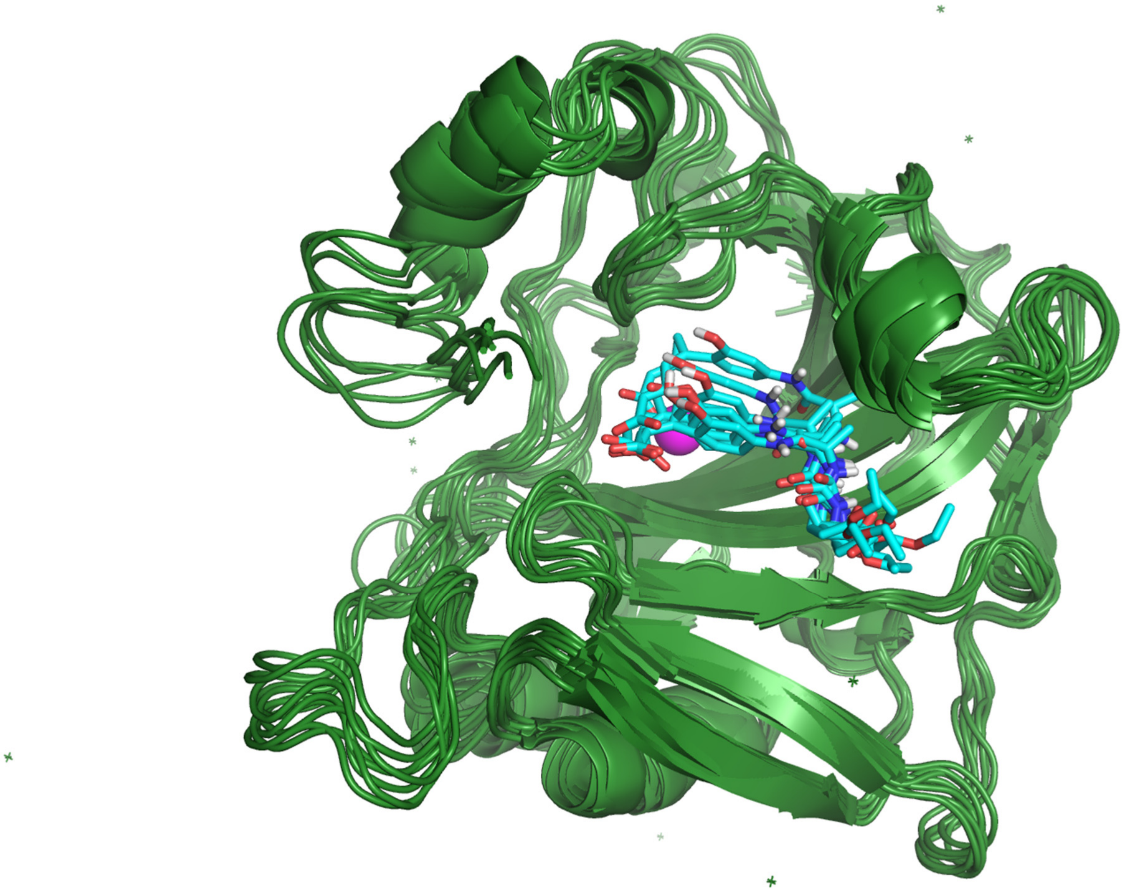

Figure S7. Alignment of CA XII:**19** representative binding geometries obtained from clustering the MD frames.

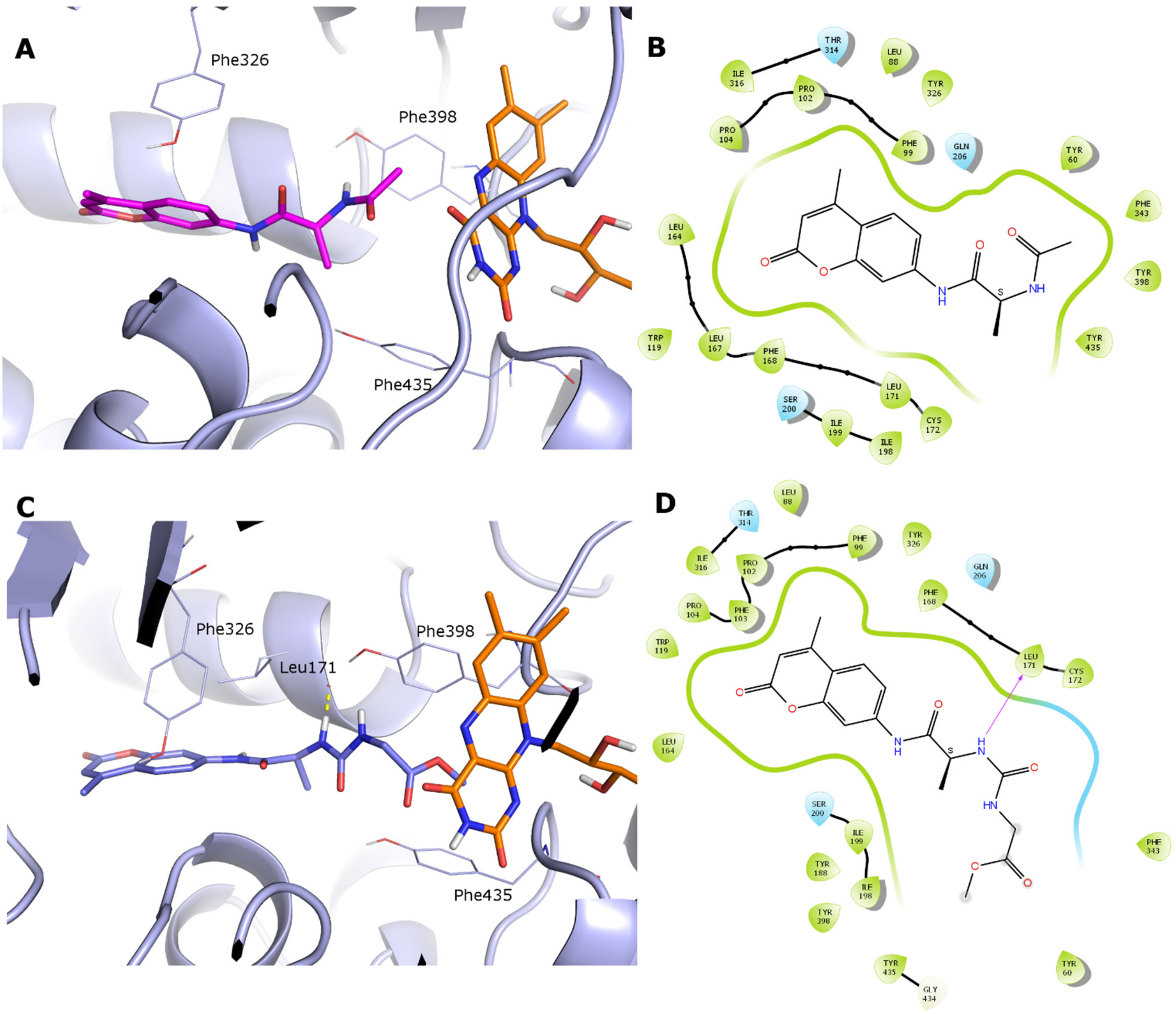

Figure S8. Panels A and C show the catalytic site of MAO-B in complex with compounds **6** and **16**, respectively. Panels B and D show the corresponding 2D interaction diagrams.

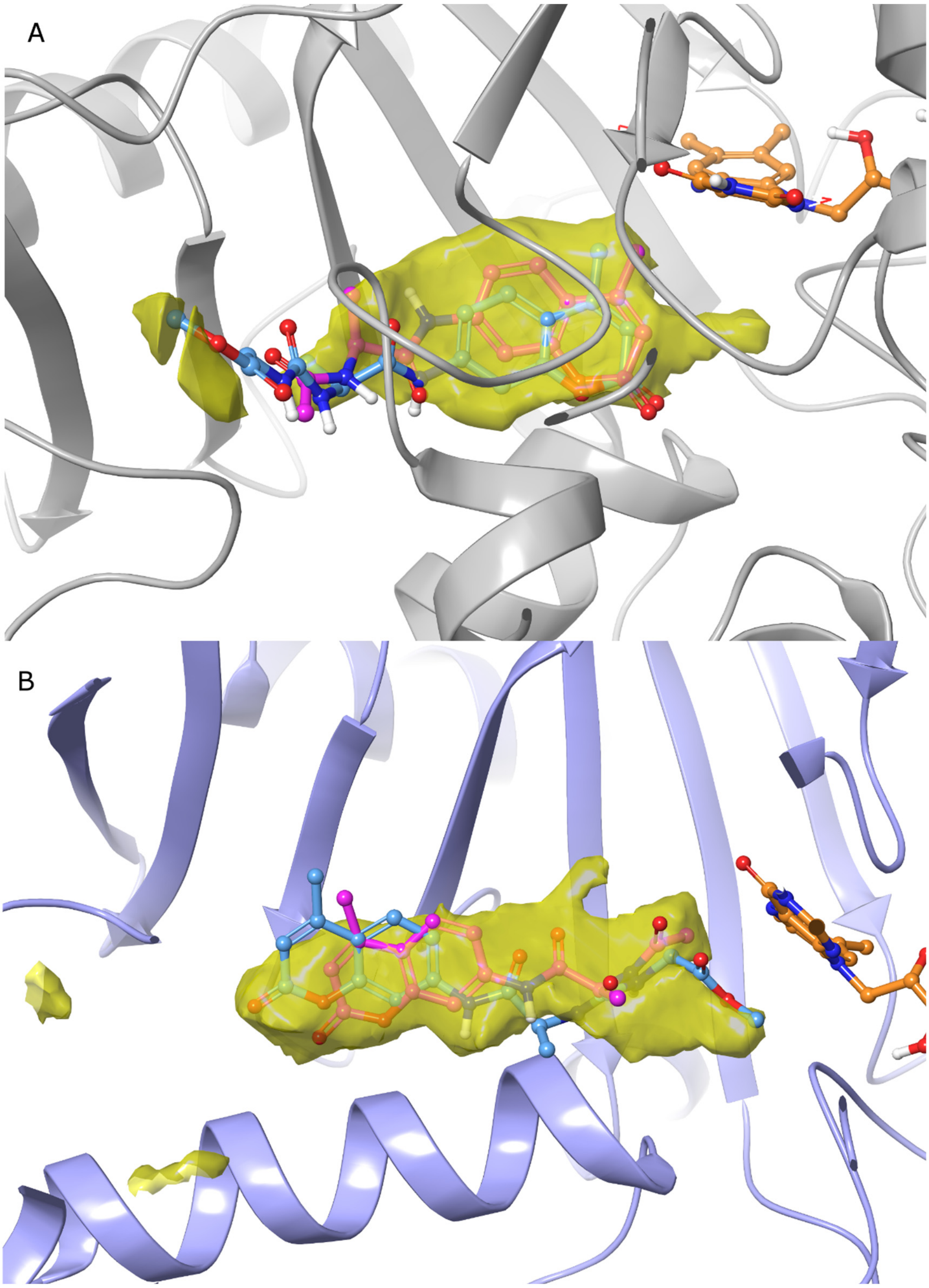

Figure S9. SiteMap calculated hydrophobic interaction region (yellow transparent surface) in the MAO-A (panel A) and MAO-B (panel B) binding site. The docked poses of ligands **6** and **16** in both enzymes are reported.

Table S1. Ligand efficiency values calculated from the inhibition data of studied coumarin derivatives against CAs and MAOs.

Compounds	hCA I	hCA II	hCA IX	hCA XII	SI	LE CA IX	LE CA XII	MAO-A	MAO-B	SI	LE MAO-A	LE MAO-B
	K_i (nM)	K_i (nM)	K_i (nM)	K_i (nM)	hCA XII			IC_{50} (µM)	IC_{50} (µM)	MAO-B/MAO-A		
Ac-Ala-MAC (**1**)	>10000	>10000	23,4	30,5	0,77	0,50	0,49	1,9195	> 100	>52,3	0,37	n/a
Z-Ala-MAC (**2**)	>10000	>10000	30,5	7,7	3,96	0,37	0,40	9,3095	> 100	>10,75	0,25	n/a
Ac-Ala-MMAC (**3**)	>10000	>10000	29,4	46,8	0,63	0,45	0,44	11,97	45,585	3,8	0,29	0,26
Z-Ile-MMAC (**4**)	>10000	>10000	78,7	41,5	1,9	0,30	0,31	46,975	> 100	>2,13	0,18	n/a
Fmoc-Ala-MAC (**5**)	>10000	>10000	91	37,2	2,45	0,28	0,29	54,8	> 100	>1,82	0,17	n/a
Z-Ala-tLeu-MMAC (**6**)	>10000	>10000	171,5	336,3	0,51	0,24	0,23	41,25	79,46	1,93	0,16	0,15
MeO-Gly-CO-Ala-MAC (**7**)	>10000	>10000	183,3	38,2	4,79	0,36	0,39	5,9095	> 100	>16,95	0,28	n/a
MeO-Ala-CO-Ala-MAC (**8**)	>10000	>10000	163,3	9,6	17,01	0,34	0,41	69,56	58,645	0,84	0,21	0,22
MeO-Phe-CO-Ala-MAC (**9**)			96,3	40		0,29	0,31	21,765	77,965	3,58	0,19	0,17
MeO-Ala-CO-Ala-MMAC (**10**)	>10000	>10000	27	54,7	0,49	0,36	0,34	> 100	75,32	<0,75	n/a	0,20
EtO-βAla-CO-Ala-MAC (**11**)	>10000	>10000	260,5	9,5	27,42	0,32	0,39	> 100	> 100	-	n/a	n/a

Table S2. Physico-chemical and pharmacokinetic properties of studied ligands calculated by QuikProp.

ID	#stars	#rotor	CNS	mol_MW	donorHB	accptHB	QPlogPo/w	QPlogS	QPlogHERG
**1**	1	3	-2	288.302	1.250	6.750	0.909	-2.579	-3.487
**2**	1	5	-2	318.329	1.250	8.450	0.749	-2.489	-3.803
**2**	1	5	-2	380.399	1.250	6.750	3.356	-5.187	-5.715
**4**	1	9	-2	452.506	1.250	8.450	3.753	-4.976	-5.491
**5**	0	5	-2	468.508	1.250	6.750	4.244	-5.520	-5.399
**6**	1	10	-2	523.585	1.500	10.200	3.190	-4.270	-4.153
**7**	1	5	-2	361.354	1.500	7.500	1.332	-4.022	-4.050
**8**	1	5	-2	375.380	1.500	7.500	1.515	-4.132	-3.726
**9**	1	5	-2	361.354	1.500	7.500	1.331	-4.020	-4.048
**10**	1	7	-2	405.407	1.500	9.200	1.355	-4.051	-4.029
**11**	0	7	-2	389.407	1.250	7.250	1.725	-2.675	-2.865

ID	QPPCaco	QPlogBB	QPPMDCK	#metab	QPlogKhsa	Percent Human Oral Absorption	PSA	Rule Of Five
**1**	192.179	-1.084	158.515	2	-0.523	73.141	111.787	0
**2**	192.286	-1.276	158.615	3	-0.717	72.208	119.403	0
**2**	285.776	-1.319	127.755	3	0.420	90.556	119.943	0
**4**	467.279	-1.353	217.371	4	0.341	96.698	123.730	0
**5**	333.991	-1.145	151.204	3	0.767	96.964	119.773	0
**6**	162.735	-1.672	123.059	5	0.051	72.248	153.820	1
**7**	58.228	-1.985	37.688	3	-0.270	66.337	158.219	0
**8**	81.188	-1.796	47.340	3	-0.209	69.994	152.593	0
**9**	58.240	-1.984	37.692	3	-0.270	66.333	158.215	0
**10**	80.980	-2.017	47.205	4	-0.404	69.037	160.233	0
**11**	68.851	-1.626	57.064	3	-0.211	69.942	153.899	0

#stars: Number of property or descriptor values that fall outside the 95% range of similar values for known drugs; rotor: number of rotatable bonds; CNS: Predicted central nervous system activity on a −2 (inactive) to +2 (active) scale; QPlogS: Predicted aqueous solubility, log S. S in mol dm^{-3} is the concentration of the solute in a saturated solution (-6.5/+0.5); QPlogHERG: Predicted IC_{50} value for blockage of HERG K^+ channels (concern below -5); QPPCaco: Predicted apparent Caco-2 cell permeability in nm/sec. Caco-2 cells are a model for the gut-blood barrier (<25 poor, >500 great); QPlogBB: Predicted brain/blood partition coefficient (−3.0/+1.2); QPPMDCK: Predicted apparent MDCK cell permeability in nm/sec. MDCK cells are considered to be a good mimic for the blood-brain barrier (<25 poor, >500 great); #metab: Number of likely metabolic reactions; QPlogKhsa: Prediction of binding to human serum albumin (-1.5/+1.5); PercentHumanOralAbsorption: Predicted human oral absorption on 0 to 100% scale; PSA: Van der Waals surface area of polar nitrogen and oxygen atoms and carbonyl carbon atoms (7-200); RuleOfFive: Number of violations of Lipinski's rule of five.
